# CTCF-dependent chromatin boundaries formed by asymmetric nucleosome arrays with decreased linker length

**DOI:** 10.1093/nar/gkz908

**Published:** 2019-10-30

**Authors:** Christopher T Clarkson, Emma A Deeks, Ralph Samarista, Hulkar Mamayusupova, Victor B Zhurkin, Vladimir B Teif

**Affiliations:** 1 School of Life Sciences, University of Essex, Wivenhoe Park, Colchester CO4 3SQ, UK; 2 Biological Sciences BSc Program, University of Essex, Wivenhoe Park, Colchester CO4 3SQ, UK; 3 Wellcome Trust Vacation Student; 4 Center for Cancer Research, National Cancer Institute, National Institutes of Health, Bethesda, MD 20892, USA

## Abstract

The CCCTC-binding factor (CTCF) organises the genome in 3D through DNA loops and in 1D by setting boundaries isolating different chromatin states, but these processes are not well understood. Here we investigate chromatin boundaries in mouse embryonic stem cells, defined by the regions with decreased Nucleosome Repeat Length (NRL) for ∼20 nucleosomes near CTCF sites, affecting up to 10% of the genome. We found that the nucleosome-depleted region (NDR) near CTCF is asymmetrically located >40 nucleotides 5′-upstream from the centre of CTCF motif. The strength of CTCF binding to DNA and the presence of cohesin is correlated with the decrease of NRL near CTCF, and anti-correlated with the level of asymmetry of the nucleosome array. Individual chromatin remodellers have different contributions, with Snf2h having the strongest effect on the NRL decrease near CTCF and Chd4 playing a major role in the symmetry breaking. Upon differentiation, a subset of preserved, common CTCF sites maintains asymmetric nucleosome pattern and small NRL. The sites which lost CTCF upon differentiation are characterized by nucleosome rearrangement 3′-downstream, with unchanged NDR 5′-upstream of CTCF motifs. Boundaries of topologically associated chromatin domains frequently contain several inward-oriented CTCF motifs whose effects, described above, add up synergistically.

## INTRODUCTION

**Figure 1.**
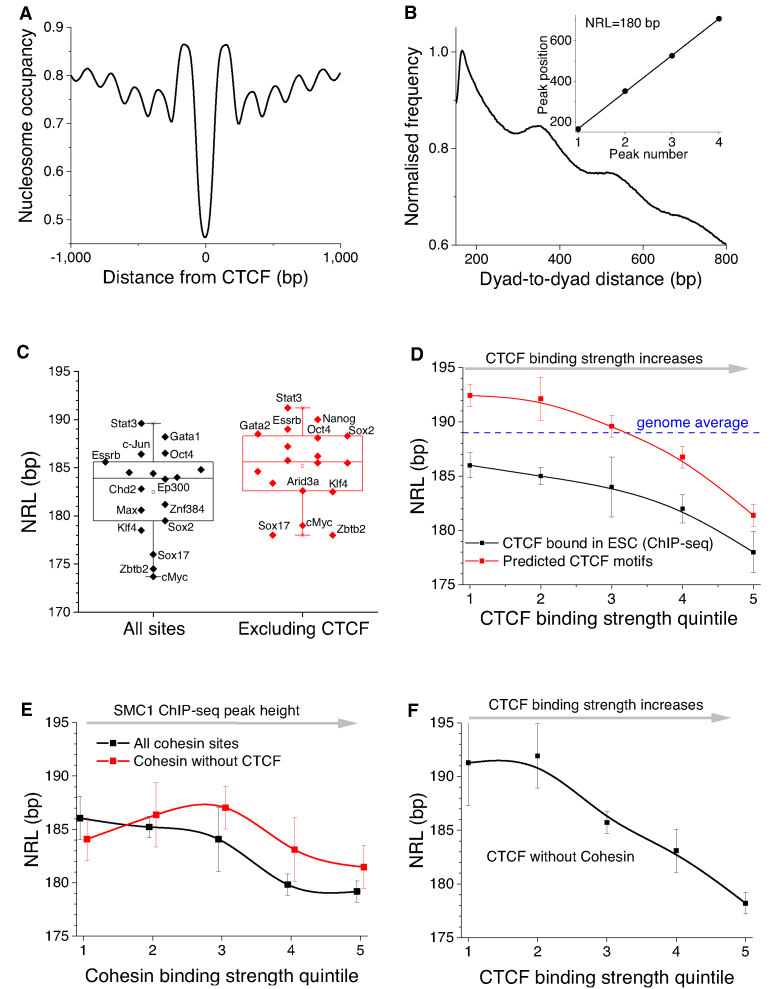
CTCF-dependent decrease of the nucleosome repeat length (NRL). (**A**) Average nucleosome profile based on MNase-seq from Voong *et al.* (42) around CTCF binding sites in ESCs determined by ChIP-seq (12). This profile is calculated without taking into account the directionality of CTCF binding. (**B**) Illustration of the ‘phasogram’ method of NRL calculation for the region [100, 2000] from the centre of experimental CTCF sites measured in ESCs. The calculation of frequencies of nucleosome dyad-to-dyad distances is followed by the linear regression of the peak positions (insert). (**C**) NRLs calculated near binding sites of 18 stemness-related chromatin proteins in ESCs in the region [100, 2000] from the summit of TF binding ChIP-seq peak, using chemical nucleosome mapping data from Voong *et al.* (42). Left: all TF binding sites; right: TF binding sites which do not intersect with CTCF. Open squares show the average NRL value based on all these TFs. The full list of experimental ChIP-seq datasets used in this calculation is provided in Supplementary Table ST1. (**D**) Dependence of NRL on the strength of CTCF binding based on experimental ChIP-seq peaks from mouse ENCODE (12) stratified into binding strength quintiles by the heights of peaks (black line) and computationally predicted CTCF sites obtained by scanning the mouse genome with TFBStools using >80% similarity for JASPAR matrix MA0139.1 stratified into binding strength quintiles by their TRAP score (red line). (**E**) NRL near bound cohesin, split into 5 quintiles based on the heights of experimental ChIP-seq peaks of the cohesin subunit SMC1 (83), calculated separately for all cohesin sites (black) and cohesin sites that do not contain CTCF motifs (red). (**F**) The same as (D), but only for experimental CTCF peaks that do not overlap with SMC1 peaks. The error bars correspond to the standard deviation of the linear fit across the peaks of the phasogram as explained in panel B.

Nucleosomes are positioned along the genome in a non-random way (1–3), which is critical for determining the DNA accessibility and genome organisation (4). A particularly important nucleosome positioning signal is provided by CTCF, an architectural protein that maintains 3D genome architecture (5–7) and can organize up to 20 nucleosomes in its vicinity (8–11) (Figure 1A). Most other TFs do not possess such nucleosome-organizing potential (Supplementary Figure S1). CTCF has ∼100 000 potential binding sites in the mouse genome. Usually there are ∼30 000–60 000 CTCF sites bound in a given cell type, which translates to about 1 million of affected nucleosomes (up to 10% of the mouse genome) (11–14). CTCF is able to act as an insulator between genomic regions with different chromatin states, but how exactly this is achieved is not known. Here, we explore molecular mechanisms of the insulator boundary formation by CTCF through rearrangement of surrounding nucleosome arrays.

One of the ways to characterize genomic nucleosome distribution is through an integral parameter called the nucleosome repeat length (NRL), defined as the average distance between the centres of adjacent nucleosomes. NRL can be defined genome-wide, locally for an individual genomic region or for a set of regions. The local NRL is particularly important, since it reflects different structures of chromatin fibres (15–19). Ever since the discovery of the nucleosome (20,21) there have been many attempts to compare NRLs of different genomic regions (22–24) and it has been established that genome-wide NRL changes during cell differentiation (25,26). Recent sequencing-based investigations showed that active regions such as promoters, enhancers and actively transcribed genes usually have shorter NRLs and heterochromatin is characterized by longer NRLs (27–30). While in Yeast it is possible to link NRL changes to the action of individual chromatin remodellers (31–35), in mouse or human regulatory regions are very heterogeneous and it is difficult to come up with a set of definitive remodeller rules determining their effect on NRL (36,37).

We previously showed that in mouse embryonic stem cells (ESC), NRL near CTCF is ∼10 bp smaller than genome-wide NRL (10,38). Our analysis demonstrated that purely statistical positioning of nucleosomes near CTCF boundaries would not be enough to explain genome-wide NRL shortening near bound CTCF observed experimentally; also, the effects of strong nucleosome-positioning DNA sequences, while compatible with the observed NRL, are limited to a small number of CTCF sites (39). A very recent study has investigated the effect of Snf2 and Brg1 remodellers on NRL in ESCs, suggesting Snf2 as the primary player (40). However, other factors may be at play as well. Thus, it is still unclear what determines the NRL near CTCF and how different CTCF sites are distinguished from each other e.g. during cell differentiation. Furthermore, recent studies have shown that CTCF can act as a boundary element between different chromatin states (e.g. DNA methylation) linearly spreading along the genome (11,41), but the mechanistic explanation for such a function is not immediately clear from the better established role of CTCF in 3D chromatin looping. Here we address these problems using available experimental datasets in ESCs and their differentiated counterparts.

We show below that the boundaries of nucleosome arrays are encoded in extended DNA regions >200 bp long enclosing individual CTCF motifs. Furthermore, the strength of CTCF binding provides a single ‘code’ that determines the value of NRL near CTCF, the level of asymmetry of CTCF-dependent nucleosome array boundaries, and eventually serves as a guide for larger-scale chromatin rearrangements during cell differentiation.

## MATERIALS AND METHODS

### Experimental datasets

Nucleosome positioning, transcription factor and chromatin remodeller binding datasets were obtained from the Gene Expression Omnibus (GEO), Short Read Archive (SRA) and the ENCODE web site as detailed in Supplementary Table ST1. NRL calculations near CTCF in ESCs were performed using the MNase-seq dataset from (42). NRL calculations near 18 stemness-related proteins in ESCs shown in Figure 1C and Supplementary Figure S3 were performed using the chemical mapping dataset from (42). NRL calculations in NPCs and MEFs were based on the MNase-seq datasets from (38). MNase-assisted H3 ChIP-seq from (11) was used for demonstrative purposes in the phasogram calculation in Figure 1B and aggregate profiles in Supplementary Figure S9. A more detailed list of datasets used in each figure is provided in Supplementary Table ST1. Coordinates of genomic features and experimental maps of transcription factor and remodeller binding in ESCs were obtained from published sources as detailed in Supplementary Table ST1. The coordinates of loops described in (43) were kindly provided by the authors in a BED file aligned to the mm10 mouse genome and converted to mm9 using liftOver (UCSC Genome Browser).

### Data pre-processing

For nucleosome positioning, raw sequencing data were aligned to the mouse mm9 genome using Bowtie allowing up to two mismatches. For all other datasets, we used processed files with genomic coordinates downloaded from the corresponding database as detailed in Supplementary Table ST1. Where required, coordinates were converted from mm10 to mm9 since the majority of the datasets were in mm9.

### Basic data processing

TF binding-sites were extended from the centre of the site to the region [100, 2000]. In order to find all nucleosomal DNA fragments inside each genomic region of interest, the bed files containing the coordinates of nucleosomes processed using the NucTools pipeline (44) were intersected with the corresponding genomic regions of interest using BedTools (45).

### Binding site prediction

Computationally predicted TF binding sites were determined via scanning the mouse mm9 genome with position frequency matrices (PFMs) from the JASPAR2018 database (46) using R packages TFBSTools (47) and GenomicRanges (48). A similarity threshold of 80% was used for all TFs in order to get at least several thousand putative binding sites. In the case of MYC, we used matrix MA0059.1 defined in *Homo sapiens*, since its matrix MA0147.2 defined in *Mus musculus* returned a significantly smaller number of sites. For all other TFs we used default JASPAR matrices provided for *Mus musculus*.

### Separation into forward and backward facing CTCF motifs

We used TFBSTools (47) to search on the 5′-3′ prime strand for forward facing CTCF motifs using the JASPAR matrix MA0139.1 and the 3′-5′ strand for motifs that are backwards facing ones. An alternative calculation using RSAT (49) with the same matrix led to similar results.

### Calculation of aggregate nucleosome profiles

Aggregate nucleosome profiles were calculated using NucTools with single-base pair resolution (44). The calculation taking into account CTCF motif directionality was done as follows: in the case, if the motif is on the plus strand, the region [−1000, 1000] near CTCF also starts left to right, whereas for the minus strand the position of the region was mirrored with respect to the middle of the CTCF site.

### Stratification of TF-DNA binding affinity

In the case of experimentally determined binding sites of CTCF, we stratified 33 880 sites, reported by the mouse ENCODE consortium (12), into five equally sized quintiles according to their ChIP-seq peak height reported in the original publication. In the case of computationally predicted TF sites, we started with 111 480 sites found by scanning the mouse genome with TFBStools using JASPAR matrix MA0139.1 with 80% similarity threshold, and split them into five equal quintiles based on their TRAP score (50). The TRAP score is proportional to the binding probability of CTCF for a given site. In order to calculate the TRAP score, we extended CTCF motifs by 30 nucleotides in both directions and used tRap implementation of the TRAP algorithm in R with default parameters (https://github.com/matthuska/tRap). In the calculations involving CTCF motif directionality (Figures 5–7) we first arranged predicted sites by the TRAP score into quintiles, and after that intersected them with the experimental ChIP-seq peaks of CTCF. Only motifs overlapping with sites that were experimentally detected by ChIP-seq in at least one mouse cell type were retained (including datasets from the mouse ENCODE project (12), GSE27944 (51), GSE96107 (43), GSE114599 (11)). These were further filtered to exclude CTCF sites separated by <1000 bp from annotated transcription start sites (TSSs), which removed about 10% of CTCF sites. TSSs were defined based on the Genomatics Eldorado database (Genomatix GmbH). After these filtering steps we obtained the following numbers of sites in the binding strength quintiles Q1−Q5: 3596 (Q1); 3782 (Q2); 6776 (Q3); 14 776 (Q4); 16 860 (Q5).

### Phasogram calculation

The ‘phasograms’ representing the histograms of dyad-to-dyad or start-to-start distances were calculated with NucTools. When paired-end MNase-seq was used, dyad-to-dyad distances were calculated using the center of each read as described previously (44). When chemical mapping data was used, this procedure was modified to use the start-to-start distances instead, because in the chemical mapping method the DNA cuts happen at the dyad nucleosome locations. The phasogram was then used for the NRL calculation as explained in Figure 1B. The NRL was defined by the slope of the line connecting the phasogram peaks; this line was determined by linear fitting, taking into account only the phasograms where ANOVA *P*-value for the slope determination is <0.05.

### Selection of the location of the region near CTCF for NRL calculations

We noticed that NRL near CTCF depends critically on the distance of the region of NRL calculation to the binding site summit (Supplementary Figure S2). While the phasograms for regions [100, 2000] and [250, 1000] near the summits of the experimental CTCF sites, which both exclude the CTCF site, are quite similar to each other, a region that includes the CTCF peak summit [−500, 500] is characterised by a very different phasogram. However, the latter phasogram is an artefact of the effect of the interference of two ‘waves’ of distances between nucleosomes: one wave corresponds to the distances between nucleosomes located on the same side of CTCF, and the second wave corresponds to distances between nucleosomes located on different sides from CTCF. The superposition of these two waves results in the appearance of additional peaks (Supplementary Figure S2A). A linear fit through all the peaks given by the interference of these two waves gives NRL = 155 bp, but this value does not reflect the real prevalent distance between nucleosomes (Supplementary Figure S2B). We thus selected the region [100, 2000] for the following calculations. Below, all NRLs refer to regions [100, 2000] near the summits of TF binding sites, unless specified otherwise. We would like to note that the effect explained above means that some of the previous publications reporting NRL near CTCF may need to be re-evaluated, because the summit of CTCF site needs to be always excluded from the genomic region for robust NRL calculations; otherwise, the apparent NRL is unrealistically small. We checked that this artefact at least does not affect NRL calculations near TSS (Supplementary Figure S2C). Once the region location with respect to the CTCF site is fixed, the phasograms are not significantly affected by the choice of the nucleosome positioning dataset (Supplementary Figure S2D). In the following calculations in ESCs we used the high-coverage MNase-seq and chemical mapping datasets from (42).

### Automated NRL determination from phasograms

Studying many phasograms proved cumbersome when manually picking the peak locations in a non-automated way. To circumvent this problem, we developed an interactive applet called *NRLcalc* based on the Shiny R framework (http://shiny.rstudio.com), to allow one to interactively annotate each phasogram such that the NRL could be calculated conveniently. *NRLcalc* allows one to select a smoothing window size to minimize noise in the phasograms. A smoothing window of 20 bp was used in our calculations. The applet also provides the *Next* and *Back* button to allow the user to go through many phasograms, as well as intuitive user interface to load and save data.

### Analysis of RNA expression near CTCF

RNA-seq data was downloaded from the GEO GSE98671 (7) and mapped with TopHat (52) to the mm9 genome. The mapped BAM files were converted to BED format with BEDOPS (53). The numbers of RNA reads aligning 1000 bp up- and downstream of CTCF motifs were calculated using BedTools (45), requiring at least 1bp intersection.

### TAD analysis

TAD coordinates in ESCs and NPCs reported by Bonev *et al.* (43) for the mm10 genome were converted to mm9 using liftOver. TADs defined as common, lost and gained upon ESC to NPC transition were determined using BedTools’ command intersect with parameter -wc. TADs with the rate of overlap between ESCs and NPCs >90% were considered common; those appearing in ESCs and NPCs with an overlap rate <80% were defined as lost and gained correspondingly. The aggregate profiles of CTCF motifs around TAD boundaries were calculated with HOMER (54) at a bin resolution of 5000 bp.

## RESULTS

### Setup of NRL calculations

Let us base our NRL calculations on the ‘phasogram’ algorithm introduced previously (27,38). The idea of this method is to consider all mapped nucleosome reads within the genomic region of interest and calculate the distribution of the frequencies of distances between nucleosome dyads. This distribution typically shows peaks corresponding to the prevalent distance between two nearest neighbour nucleosomes followed by the distances between next neighbours. The slope of the line resulting from the linear fit of the positions of the peaks then gives the NRL (Figure 1B). To perform bulk calculations of NRLs for many genomic subsets of interest we developed software *NRLcalc*, which loads the phasograms computed in *NucTools* (44) and performs linear fitting to calculate the NRL (see Materials and Methods).

Each TF is characterised by a unique NRL distribution near its binding sites. For example, we used a recently reported chemical nucleosome mapping dataset (42) to calculate NRLs in the region of up to 2000 bp from the centre of the binding site excluding the central 100 bp (hereafter referred to as region [100, 2000]) for 18 stemness-related TFs whose binding has been experimentally determined in ESCs using ChIP-seq (Figure 1C). This analysis revealed that the proximity to CTCF binding sites unanimously reduced the NRL near these sites. When we filtered out TF binding sites that overlap with CTCF binding sites in ESCs, the NRLs for each individual TF increased (Figure 1C). On the other hand, TF binding sites that overlap with CTCF had significantly smaller NRLs (Supplementary Figure S3).

### The strength of CTCF binding correlates with NRL decrease in the adjacent region

To dig deeper into the relationship between CTCF and local chromatin conformation, we split CTCF sites into 5 quintiles of increasing binding strength. Two metrics were used as a means of quantifying CTCF binding strength: (i) Experimentally determined CTCF binding sites in ESCs were split into five quintiles based on the height of the ChIP-seq peaks reported by the mouse ENCODE consortium (12). (ii) Theoretically predicted binding sites defined by scanning the mouse genome using TFBStools (47) with the 19-bp CTCF motif (JASPAR MA0139.1) (46) were split into five quintiles based on their calculated TRAP score that is proportional to the probability of CTCF binding to a given site (50) (see Materials and Methods). In each case, the calculation of the NRL was performed in the region [100, 2000] near CTCF binding sites using MNase-seq data (42). These calculations revealed a smooth decrease of NRL as the strength of CTCF binding increased in the case of both used metrics (Figure 1D). In addition, we used the chemical nucleosome mapping dataset (42) to compare the CTCF quintiles in terms of the distribution of nucleosome dyad-to-dyad distances, which also revealed that stronger CTCF binding is associated with smaller NRLs (Supplementary Figure S4). Thus, the CTCF-dependent NRL decrease is a general, dataset-independent effect. Note that chemical mapping-based NRLs should not be directly compared with MNase-seq ones due to the inherent peculiarities of the chemical mapping experiment that we noticed previously (44); below we will use only MNase-seq and MNase-assisted histone H3 ChIP-seq datasets for nucleosome mapping.

We then asked, whether the same effect on NRL is observed for CTCF’s binding partner cohesin. Cohesin is a ring-shaped complex that slides along one or two DNA double helices until it meets CTCF, thus extruding DNA loops (55). Cohesin is able to induce regular nucleosome arrays around it even when not associated with CTCF (Supplementary Figure S5), thus it is interesting whether it has a similar effect on NRL. Cohesin does not have its own DNA sequence preferences, but we can still stratify mapped cohesin locations in terms of the strength of binding using ChIP-seq of cohesin's component SMC1 and sorting its occupancy peaks into quintiles based on their height. Figure 1E shows that, similarly to CTCF, cohesin sites are characterized by the local NRL decrease as cohesin's binding strength increases. However, the effect of cohesin's binding strength on NRL is weaker than that for CTCF, and almost disappears if only the cohesin sites that do not contain CTCF motifs are considered (Figure 1E). On the other hand, the bound CTCFs that do not overlap with bound cohesin in ESCs still display a pronounced effect of CTCF binding strength on NRL (Figure 1F). This effect was also recapitulated for CTCF sites residing at least 10 000 bp outside of annotated TSSs (Supplementary Figure S6), showing that it was not caused by protein coding gene transcription.

**Figure 2.**
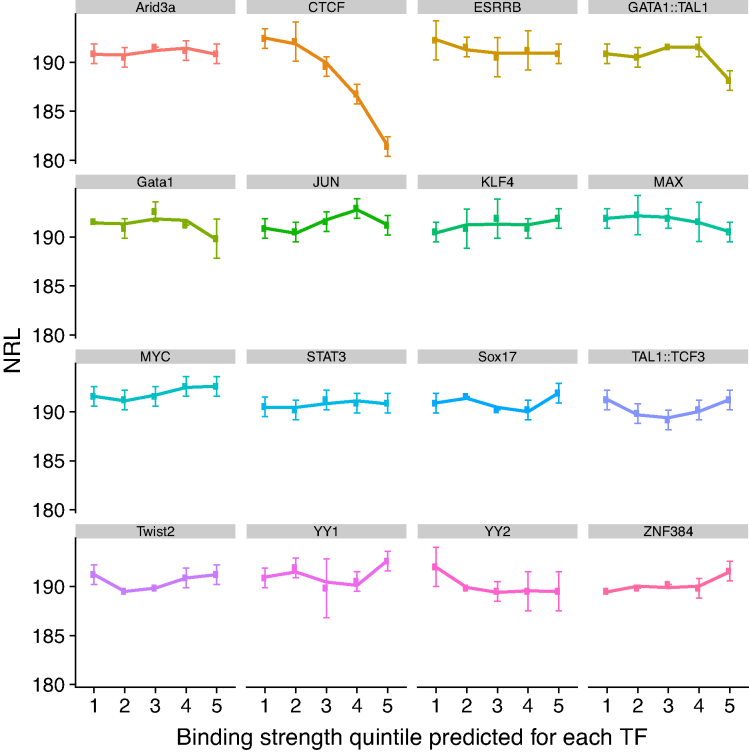
Proteins other than CTCF and cohesin do not show the relationship between DNA-binding strength and NRL near their binding sites. Sixteen representative TFs related to stem cells are shown (similar calculations were performed for 497 TFs listed in JASPAR2018). TF bindings sites used in this analysis were predicted computationally by scanning the mouse genome using TFBStools with the 80% motif similarity cut off and then stratified into five binding strength quintiles based on the TRAP score (see Materials and Methods).

Using the same procedure we have investigated NRL near other chromatin proteins. Firstly, we considered 497 TFs which have position weight matrices in JASPAR2018 (46), and for each of them calculated NRL in the region [100, 2000] from the TF motif as a function of the DNA-binding strength predicted for a given TF. This analysis revealed that for TFs other than CTCF, the NRLs did not reveal a monotonic function of their binding strength (see Figure 2 for examples of TFs relevant to stem cells). We have also performed a similar calculation for chromatin remodellers that have been experimentally profiled in ESCs, asking whether NRL in the region [−1000, 1000] near remodeller depends on the height of the corresponding remodeller peak (Supplementary Figure S7). These calculations did not reveal NRL dependence on the binding strength as in the case of CTCF or cohesin. Thus, CTCF and cohesin are unique proteins whose DNA binding strength is anticorrelated to the NRL value.

### The strength of CTCF-DNA binding correlates with GC and CpG content

**Figure 3.**
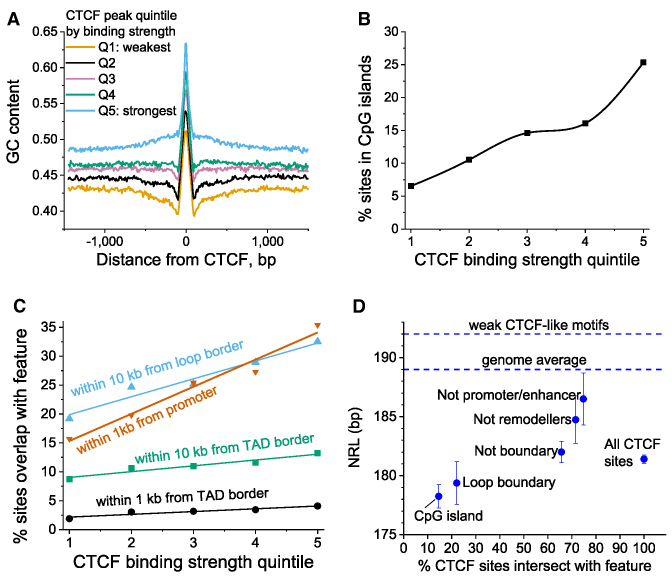
Genetic features correlating with the experimental strength of CTCF binding. (**A**) CTCF binding sites split into quintiles based on their binding strength are characterized by increasing GC content as CTCF binding strength increases. (**B**) The stronger CTCF binding site the higher is the probability that it is located in a CpG island. (**C**) The stronger CTCF binds the higher the probability that it is located in a promoter or forms a boundary of TADs or enhancer-promoter loops. (**D**) NRLs for the following subsets of CTCF sites: all sites bound in ESCs; inside chromatin loop boundary; outside of boundaries of loops and TADs; inside CpG islands; outside of chromatin remodeller peaks; outside of promoters and enhancers. The top horizontal dashed line corresponds to the weak CTCF-like motifs from Figure 1D. Vertical bars show the standard deviation.

In order to understand the physical mechanisms of NRL decrease near CTCF, we considered a number of genomic features and molecular factors that could potentially account for the NRL decrease near CTCF (Figure 3). A number of previous observations suggested that the ability of CTCF sites to retain CTCF during cell perturbations is related to the surrounding GC and CpG content (11,56). Our calculations performed here provide more detail on this effect, showing that the strength of CTCF binding is correlated with GC content around CTCF sites (Figure 3A), and that the probability for a given site to be located in a CpG island monotonically increases with the CTCF binding strength (Figure 3B). It is worth noting that the CTCF motif itself is GC-rich, which corresponds to the central peak in Figure 3A, but the effects mentioned above extend to distances >1000 bp from CTCF motif. Furthermore, the CTCF site location inside CpG islands was associated with a significantly decreased NRL in comparison with all CTCF sites (Figure 3D).

### The strength of CTCF-DNA binding correlates with the probability of a given site to be inside *cis*-regulatory elements and domain boundaries

Another potential hypothesis is that the small NRL near CTCF could be because CTCF sites are in active regions (promoters, enhancers, etc.) which have a smaller NRL in comparison to genome-average based on previous studies (27,28). Our analysis performed here demonstrated that there is a positive correlation between the strength of CTCF binding and the probability that it is inside a promoter region (Figure 3C). We also used recently published coordinates of topologically associated domains (TADs) and promoter-enhancer loops in ESCs (43) and showed that there is a correlation between the strength of CTCF binding and the probability that it forms a boundary of TADs and even higher correlation for the boundaries of loops (Figure 3C). Furthermore, the NRL near CTCF sites was smaller if these sites were inside borders of loops or TADs, while the NRL value went up if all known regulatory regions were excluded (Figure 3D).

### Remodeller-specific effects on NRL near CTCF

**Figure 4.**
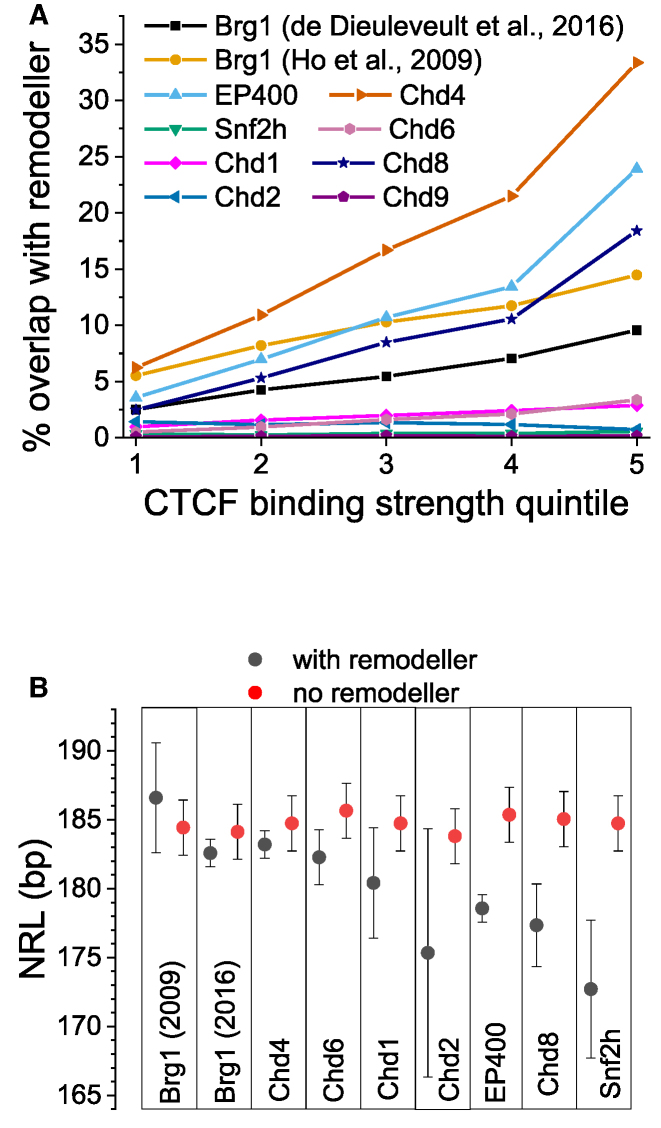
Effects of different chromatin remodellers on the value of NRL near CTCF. (**A**) The stronger CTCF binds the higher is the probability that it is co-enriched with different chromatin remodellers indicated on the figure. The enrichment was defined as the ratio of CTCF sites overlapping with ChIP-seq peaks of a given remodeller to the total number of CTCF sites in a given quintile. (**B**) NRLs calculated near CTCF sites that overlap (black) and do not overlap (red) with ChIP-seq peaks of eight chromatin remodellers experimentally mapped in ESCs. Remodeller names are indicated on the figure. Two Brg1 datasets reported in 2009 and 2016 are taken from separate publications, (84) and (36) respectively.

Active nucleosome positioning is determined by chromatin remodellers, but the rules of action of individual remodellers are not well defined. In order to clarify remodeller effects on NRL decrease near CTCF, we processed all available remodeller ChIP-seq datasets in ESCs and plotted the percentage of CTCF sites overlapping with remodeller ChIP-seq peaks (Figure 4A). This analysis showed that the stronger CTCF binds the higher the probability that a given CTCF binding site overlaps with remodellers. Particularly large percentage of CTCF sites overlaps with peaks of remodellers Chd4, EP400, Chd8 and BRG1, with Chd4 being the top CTCF-related remodeller. We have also performed similar analysis for three different TFs: CTCFL, Oct4 and c-Jun (Supplementary Figure S8). CTCFL (also known as BORIS), shares a number of sites with CTCF, and unsurprisingly BORIS and CTCF have similar preferences for remodellers. On the other hand, Oct4, which is highly expressed in ESCs, showed a qualitatively similar effect of increasing co-binding with remodellers as its DNA sequence-determined binding strength increases, but the top Oct4-associated remodeller was BRG1 rather than Chd4. As a negative control, we considered c-Jun, which is not a stem cell TF. As expected, for c-Jun binding sites the percentage of intersection with remodeller peaks did not depend on the predicted strength of c-Jun binding to DNA (Supplementary Figure S8).

Next we set to derive systematic rules of remodeller effects on NRL near CTCF (Figure 4B). By comparing NRLs near CTCF sites overlapping and non-overlapping with each remodeller, we learned that Brg1 has no detectable effect (based on two independent Brg1 datasets), and Snf2h has the strongest effect. The effect of other remodellers on NRL near CTCF is increasing in the order BRG1 ≤ Chd4 < Chd6 < Chd1 ≤ Chd2 ≤ EP400 ≤ Chd8 < Snf2h (Figure 4B).

### CTCF motif directionality introduces asymmetry in adjacent nucleosome distribution

All our calculations above were performed without considering the directionality of the CTCF motif. For example, Figure 1A shows a symmetric pattern of nucleosome occupancy around CTCF, which arises due to averaging of different patterns around CTCF motifs in the direction of the plus and minus strand. Now let us always orient the CTCF motif in the same way, left to right (5′ to 3′), and refer to positions in 5′ direction from the CTCF motif as ‘upstream’ and 3′ direction as ‘downstream’. Using this setup, we calculated aggregate profiles of nucleosome occupancy around CTCF by aligning all regions in 5′ to 3′ direction of the CTCF motif defined by the JASPAR matrix (MA0139.1). In these calculations we considered only CTCF motifs located in ChIP-seq defined peaks in at least one mouse cell type. Furthermore, we excluded CTCF sites that are located inside annotated promoters (see Materials and Methods).

**Figure 5.**
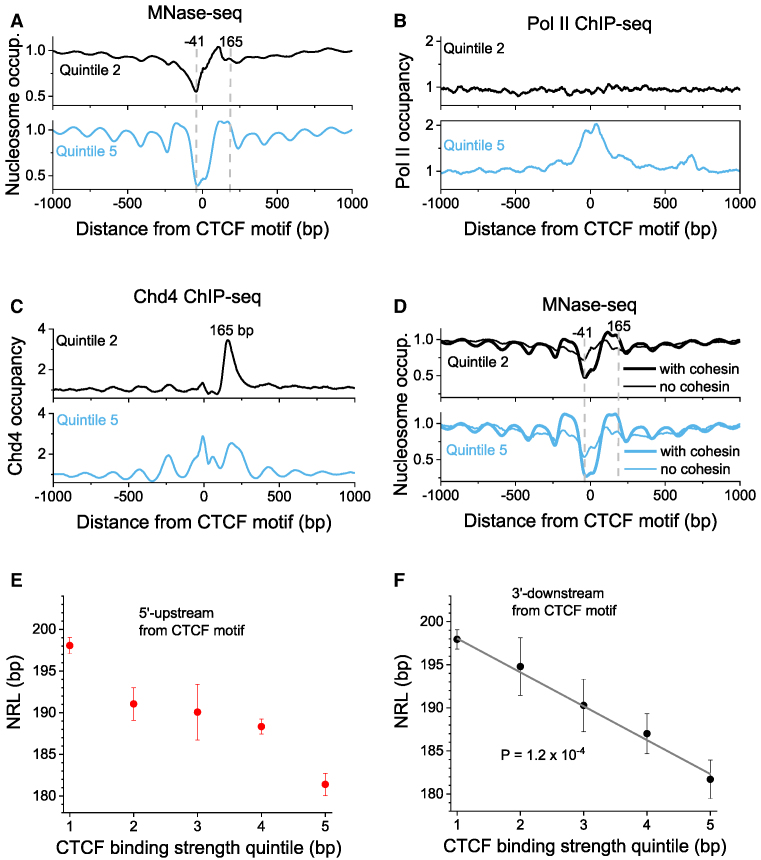
Combined effects of CTCF motif directionality and binding strength on nucleosome positioning. (**A**) Aggregate nucleosome profiles based on MNase-seq (42) around CTCF motifs outside promoters which coincide with experimentally verified binding sites in at least one mouse cell types, taking into account the DNA strand directionality. The strong peak at 105 bp from the centre of CTCF motif appears for all CTCF quintiles. On the other hand, the nucleosome peak at position 165 is sensitive to the strength of CTCF binding and increases as the strength of CTCF binding increases from weak binding at quintile 2 to strong binding at quintile 5. (**B**) CTCF binding outside of promoters is associated with CTCF-dependent Pol II enrichment. In the weakest CTCF quintile there is no Pol II enrichment, so the promoter-like nucleosome occupancy near CTCF is not due to Pol II. (**C**) The binding of Chd4 (and not any other experimentally profiled remodeller) shows a CTCF dependent peak at 165 bp, coinciding with the nucleosome occupancy peak. (**D**) Nucleosome positioning based on MNase-seq, as in panel A, but CTCF sites are split into those that overlap with the cohesin subunit SMC1 (thick line) and do not overlap with SMC1 (thin line). E and F) NRL as a function of CTCF binding strength quintile corrected for the CTCF motif directionality. (**E**) NRL calculated in the region [−2000, 100] in 5′ direction (‘upstream’) of the centre of CTCF motif. (**F**) NRL calculated in the region [100, 2000] in 3′ direction (‘downstream’) of the centre of CTCF motif. In the latter case NRL dependence of CTCF binding strength can be fitted as a straight line (*t*-test *P* = 1.2 × 10^−4^).

Figure 5A shows the aggregate profiles of MNase-seq nucleosome occupancy (42) around CTCF in ESCs taking into account the motif directionality. Here, the wave-like pattern of the nucleosome occupancy around CTCF sites reveals strong asymmetry. To the best of our knowledge this is the first report of such a pronounced nucleosome asymmetry around CTCF motifs. Counterintuitively, the weaker the CTCF binding, the stronger is the asymmetry. Such an asymmetry is similar to what is usually observed near promoters, except that we have excluded from this calculation CTCF sites that overlap with promoters. We have also confirmed this effect using MNase-assisted H3 ChIP-seq dataset (Figure S9) and plotted the occupancy of RNA Pol II around CTCF (Figure 5B). Pol II occupancy shows CTCF-dependent enrichment, which increases with the increase of CTCF binding strength. Weak CTCF sites which have the strongest asymmetry are devoid of Pol II. Thus, the asymmetry of nucleosome occupancy near CTCF is not due to Pol II-dependent transcription.

The most striking feature of the asymmetric nucleosome profiles near CTCF is that the deepest point of the nucleosome-depleted region is shifted about 41 bp ‘upstream’ in 5′ direction from the centre of the CTCF motif. This is different from what is usually assumed based on symmetric profiles such as in Figure 1A. Interestingly, the first strong nucleosome peak at 105 bp ‘downstream’ in 3′ direction from CTCF appears similarly for all CTCF site quintiles, whereas the next peak at 165 bp ‘downstream’ in 3′ direction from CTCF is extremely sensitive to the CTCF binding strength. There are also several other nucleosome occupancy peaks that display strong sensitivity to the CTCF binding strength.

### The CTCF-dependent peak of nucleosome occupancy 3′-downstream of CTCF can be attributed to Chd4

In order to determine the structural origin of the nucleosome occupancy peak at 165 bp from the CTCF, motif we calculated aggregate profiles of all chromatin remodellers using their ChIP-seq binding datasets in ESCs (Supplementary Figure S10). Interestingly, Figure S10 shows that the remodellers position themselves between nucleosomes. Chd4 is the only remodeller characterized by a CTCF-dependent peak at position +165 bp (Figure 5C). The peak of Chd4 at this location is quite pronounced, which is consistent with Chd4 being the top CTCF-associated remodeller (Figure 4A). Thus, Chd4 plays an important role in establishing the asymmetry of nucleosome positioning, while it does not affect the NRL value *per se* (Figure 4B). On the other hand, another remodeller Snf2h affects the value of NRL and the regularity of the nucleosome near CTCF (see Supplementary Figure S11, plotted using the recent Snf2h knockout data (40)).

### CTCF creates asymmetric nucleosome arrays; cohesin symmetrises them

Next we investigated the interplay between CTCF and cohesin in relation to the asymmetry of nucleosome arrays. Cohesin's subunits Rad21 and SMC1 bind quite symmetrically with respect to the CTCF motif (Supplementary Figure S12) and they have a dramatic effect on the symmetry of nucleosome arrays around CTCF (Figure 5D). Our calculations showed that for all CTCF binding strength quintiles, CTCFs which are not co-bound with cohesin create asymmetric and less regular nucleosome arrays, whereas CTCFs co-bound with cohesin create more symmetric and more regular arrays of nucleosomes (Figure 5D).

### The value of NRL in the region 3′-downstream of the CTCF motif linearly depends on the CTCF binding strength

The effect of CTCF motif directionality introduces a significant correction to the NRL dependence on the CTCF binding strength that we found above (Figure 5E and F). When performing NRL calculations separately for the region [100, 2000] 3′-downstream and region [−2000, −100] 5′-upstream from the centre of the CTCF motif, we noticed that the most regular behaviour is observed 3′-downstream, where the effect can be described by a linear dependence (Figure 5F). We also checked whether the appearance of the nucleosome occupancy peak 165 bp downstream of CTCF is the main determinant of the NRL decrease. The recalculation of the NRL in the interval [300, 2000] 3′-downstream from CTCF showed that while the NRL decrease is less steep, it still follows the same trend (Supplementary Figure S13).

### The asymmetric nucleosome depletion 5′-upstream of CTCF/CTCFL motifs is encoded in DNA repeats and may be linked to their transcription

**Figure 6.**
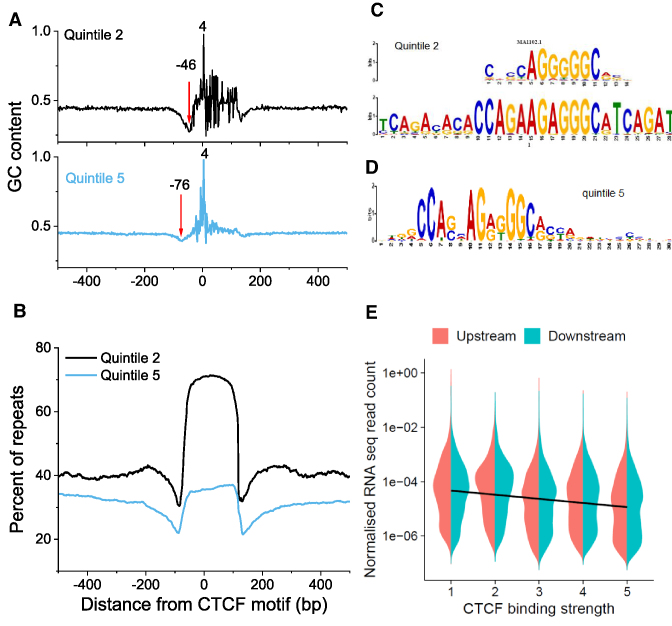
Effects of the nucleotide content around CTCF sites. (**A**) Average GC content around CTCF motifs for CTCF binding strength quintiles 2 and 5. (**B**) The percentage of repeats determined by the USCS Genome Browser's Repeat Masker as a function of the distance from the middle of CTCF motifs. (**C**) The sequence of the consensus motif in quintile 2 with the smallest *P*-value. The best TF match for the quintile 2 consensus motif is CTCFL (Boris) (JASPAR MA1102.1). (**D**) The sequence of the consensus motif in quintile 5. The quintile 5 consensus sequence contains the classical CTCF motif (JASPAR MA0139.1). (**E**) Violin plot showing the numbers of RNA reads expressed from the regions [−1000; 0] and [0; 1000], respectively upstream (red) and downstream (blue) of CTCF binding sites, as a function of CTCF binding strength. The straight line is a linear fit through all the points, showing a general decrease of the number of RNA reads as CTCF binding strength increases (*P* = 1.2e−11). The linear fits performed separately across ‘downstream’ or ‘upstream’ regions are not distinguishable.

Next we calculated the average nucleotide distribution around CTCF sites used above taking into account the orientation of CTCF motifs. This revealed an unexpected nucleotide pattern in the extended region near CTCF (Figure 6A). The nucleosome depletion in the region around −41 bp upstream of CTCF is associated with a decrease of GC content. This is consistent with previous observations that high AT-content and in particular poly(dA:dT)-tracts have strong nucleosome-excluding properties (57). It is worth noting that the CTCF motif used in our calculations is just 19 bp, but the length of the highly structured area near CTCF is more than 200 bp. This means that the CTCF motif is frequently encountered as part of a much larger DNA sequence organisation, some type of sequence repeats that are primarily responsible for the establishment of the asymmetric boundaries around CTCF. Indeed, 50% of the CTCF motifs used in our calculations in Figures 5 and 6 overlapped with repeats defined by the UCSC Genome Browser repeat masker. Furthermore, the percentage of repeats given by the repeat masker shows a very structured profile with an extended region (>200 bp) near CTCF strongly enriched with repeats (Figure 6B).

Another interesting finding shown in Figure 6C and D is that when we subjected each binding strength quintile to a separate *de novo* motif discovery, the strongest quintile 5 was associated with the classical CTCF motif (JASPAR MA0139.1), whereas a weak quintile 2 was associated with CTCFL (BORIS) defined by the JASPAR matrix MA1102.1. To the best of our knowledge this is the first indication that CTCF and CTCFL may have different effects on nucleosomal organisation (Figure 5A).

We have also checked whether the nucleosome depletion 5′-upstream of CTCF is related to transposon transcription. Using coordinates of ChIP-seq peaks of RNA Pol III determined previously in ESCs (58), we found that 33% of co-localisations of TFIIIC and Pol III and 17% of co-localisations of SINE repeats and Pol III overlapped with our CTCF motifs. Thus, not only the DNA repeats are responsible for the AT-rich region 5′-upstream of CTCF, but also their Pol III-dependent transcription may be linked to the asymmetric nucleosome depletion pattern.

### CTCF binding directly affects expression of adjacent RNA

In order to investigate quantitatively the effect of CTCF on RNA expression, we plotted the normalized amount of total RNA reads within [−1000, 1000] from CTCF as a function of CTCF binding strength (Figure 6E). It showed that the strong CTCF binding correlates with the weaker expression of neighbouring RNA (*P* = 1.2e−11). There was no significant asymmetry in RNA expression up- or downstream of the CTCF motif.

### Nucleosome-depleted boundaries 5′-upstream of CTCF motif are preserved even if binding CTCF is lost during cell differentiation

**Figure 7.**
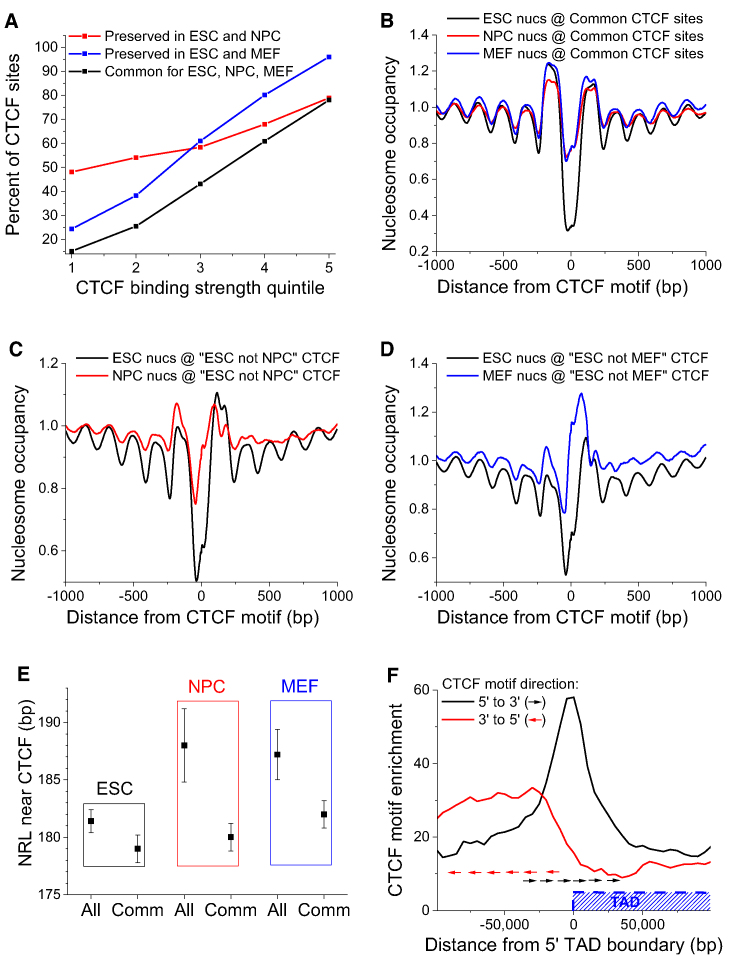
Effects of asymmetric CTCF-dependent boundaries in stem cell differentiation. (**A**) The fraction of CTCF sites preserved upon differentiation of ESCs to NPCs and MEFs as a function of CTCF binding strength. CTCF sites preserves in all these three cell types are termed ‘common’. (**B**) Nucleosome occupancy in ESCs (black), NPCs (red) and MEFs (blue) around CTCF sites common between ESC, NPC and MEF, calculated taking into account CTCF motif directionality. (**C**) Nucleosome occupancy around ‘ESC not MEF’ sites that are present in ESCs (black line) but lost in MEFs (red line) taking into account CTCF motif directionality. (**D**) Nucleosome occupancy around ‘ESC not NPC’ sites that are present in ESCs (black line) but lost in NPCs (red line) taking into account CTCF motif directionality. Note that in differentiated cells a nucleosome is being positioned to cover the ‘lost’ CTCF sites, but nucleosome depletion on the left of CTCF is still preserved. (**E**) NRLs in region [100, 2000] from CTCF’s experimental binding site summit calculated without taking into account the motif directionality. Upon differentiation average NRL near CTCF increases (denoted ‘All’), but common CTCF sites keep the smallest NRL (denoted ‘Comm’). (**F**) Enrichment of the strongest CTCF motifs (5th quintile) near 5′-boundaries of TADs in ESC, calculated separately for CTCF motifs oriented 5′-to-3′ (black) and 3′-to-5′ (red). The TAD is located to the right from the 5′-boundary. The arrows show an example of CTCF motif distribution for an individual region.

Next we compared nucleosome positioning around CTCF motifs upon differentiation of ESCs to neural progenitor cells (NPCs), as well as in the differentiated mouse embryonic fibroblasts (MEFs) using MNase-seq data from (38) and CTCF ChIP-seq data from (12,43) (Figure 7A). Notably, stronger CTCF binding to DNA increases the probability that a given site will remain bound upon differentiation. This suggests that the sequence-dependent strength of CTCF binding can act as the ‘CTCF code’, determining which CTCF sites are retained and lost upon differentiation (and thus how the 3D structure of the genome will change). Our further analysis revealed that common CTCF sites that are present in all three states are characterized by quite minor asymmetry of nucleosome organisation (Figure 7B). On the other hand, CTCF sites that are lost upon ESC differentiation to NPCs and MEFs have more profound asymmetry of the nucleosome pattern around them (Figure 7C and D). Upon differentiation both in NPCs and MEFs, the array of nucleosomes 3′-downstream of the CTCF motif is shifted to cover the CTCF site. It is worth noting that nucleosome positioning in this region is only partly CTCF-dependent. For example, inside the [−100, 100] region around CTCF, the percentage of nucleosomes covering the CTCF motifs that lost CTCF upon differentiation changes from 47% to 60% upon ESC to MEF transition, and from 42% to 54% upon ESC differentiation to NPC. Interestingly, the nucleosome-depleted region 5′-upstream of CTCF still remains open upon differentiation. The latter effect was also confirmed for the case of CTCF sites that are not bound by CTCF in ESCs and become bound in MEFs (Supplementary Figure S15).

### Common CTCF sites preserve local nucleosome organisation during ESC differentiation

Then, we set to determine the functional consequences of the NRL decrease near CTCF. NRL near bound CTCF on average increases as the cells differentiate from ESCs to NPCs or MEFs (Figure 7E and Supplementary Figure S16). However, common CTCF sites resist this NRL change, suggesting that CTCF retention at common sites upon differentiation preserves both 3D structure and nucleosome patterns at these loci. As we have established previously (Figure 5F), the effect of the active CTCF-dependent NRL decrease is mostly pronounced in the region 3′-downstream of CTCF motifs. The NRL increase near CTCF upon cell differentiation is also mostly in the 3′-downstream region (Supplementary Figure S17).

### Directed CTCF motifs mark TAD boundaries

Our previous calculations were performed at the level of boundaries formed by single CTCF motifs. However, in some cases chromatin boundaries are created by cumulative action of several CTCF motifs located not far from each other. In particular, our calculations showed that CTCF motifs oriented toward the inner part of TAD are centred at the TAD boundaries, whereas the outward-looking CTCF motifs are enriched at the outer side of the boundaries (Figure 7F). TADs that were lost upon differentiation demonstrate a smaller enrichment of CTCF motifs near them (Supplementary Figure S18), which suggests that CTCF motifs at functionally important chromatin boundaries may act additively. Thus, the effects of individual CTCF motifs described above can be summed up at a region of up to several kb, to act synergistically at the boundaries between large chromatin domains.

## DISCUSSION

**Figure 8.**
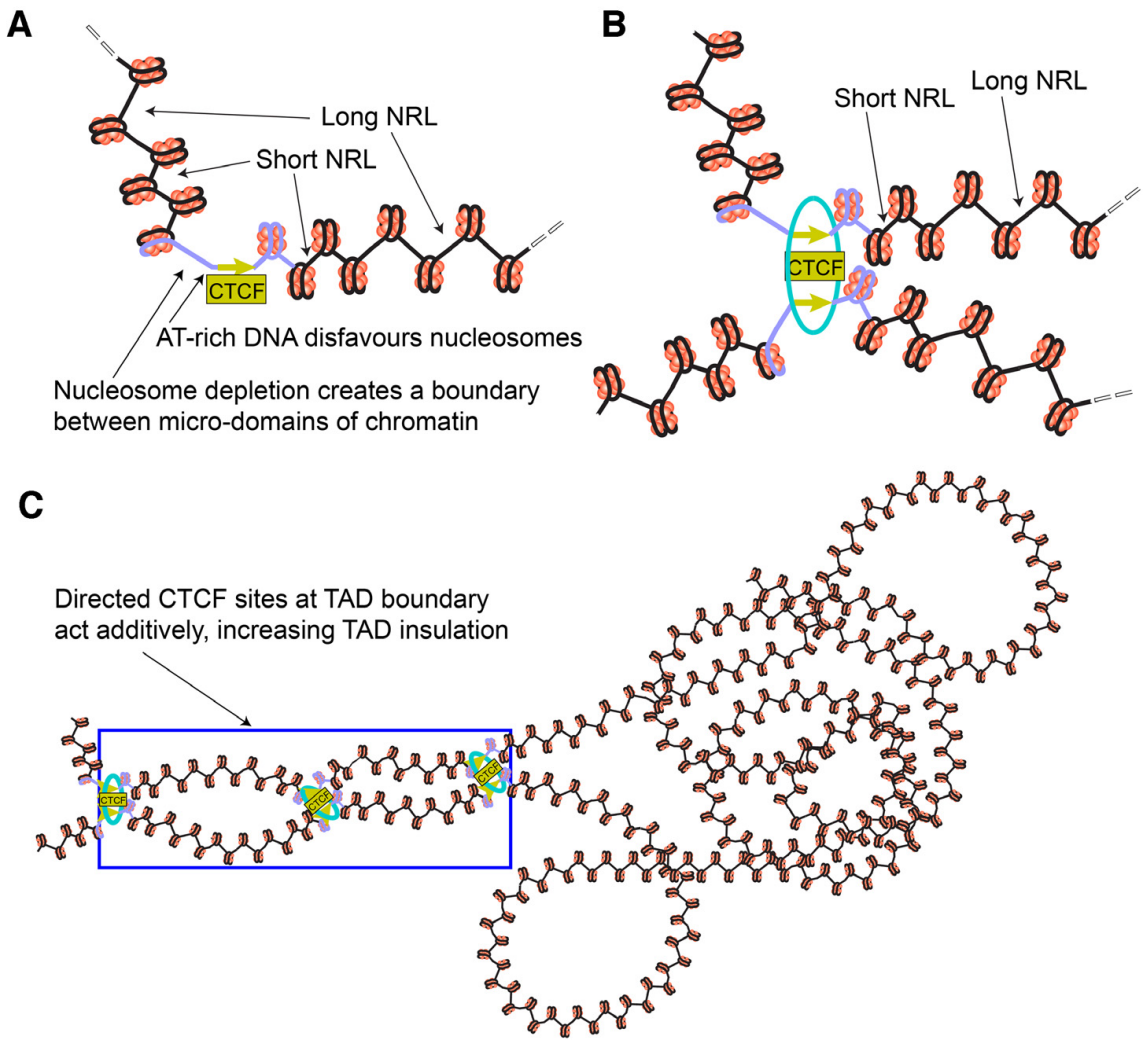
Schematic illustration of the effect of CTCF binding strength and motif orientation on the nucleosome arrangement in a single genomic region (**A**), at the base of a loop (**B**), and as part of a chromatin boundary containing several CTCF motifs (**C**). An extended DNA region including CTCF motif is enriched with repetitive sequences that define the mechanical properties of this region as a chromatin boundary (shown in violet colour)—see Figures 5A, 6A, D and Supplementary Figure S4. The region 5′-upstream of CTCF motif contains AT-rich sequences that disfavour nucleosome formation and may account for DNA bending in the complex with CTCF. Such regions can be due to DNA repeats such as SINEs, some of which are transcribed by Pol III that interact with CTCF. In analogy to the coding gene transcription the region 5′-upstream of the CTCF motif is depleted of the ‘-1’ nucleosome. In the region 3′-downstream of CTCF motif chromatin remodellers including Chd4 and Snf2h determine the regularity of the nucleosome array. The nucleosomes located close to CTCF are separated by shorter linkers and nucleosomes further away from CTCF are separated by longer linkers, reaching the genome-average linker length at distances where CTCF effects disappear (corresponding to NRL change from ∼180 bp near strong CTCF sites to ∼190 bp genome-average, see Figure 3D). The cohesin ring is represented by the cyan ellipse. In the chromatin boundary containing several CTCF motifs, the effects described above for individual CTCFs may add up to increase chromatin domain insulation through the construction of special nucleosome array packing at the boundary, physically preventing interactions between adjacent TADs.

We developed a new *NRLcalc* methodology to investigate nucleosome rearrangement and NRL changes near TF binding motifs distinguished by their orientation and binding strength. The application of this method to CTCF and cohesin binding sites revealed a number of new effects (Figure 8):

Firstly, we found that contrary to previous assumptions, the nucleosome arrangement near CTCF motifs is asymmetric and to a large degree hard-wired in the sequence of the DNA region >200 bp long including the CTCF motif (Figures 5A and 6A). The asymmetry in this case is not just a consequence of heterogeneity of nucleosome distributions around subsets of sites (59), but is a generic feature across all CTCF sites. The nucleosome-depleted region, which was previously believed to coincide with the CTCF binding site (10,39), is actually shifted 5′-upstream of CTCF motif (Figure 5). This nucleosome depletion is associated with AT-rich DNA sequence repeats which may disfavour nucleosome formation (57) and introduce bending of the double helix near CTCF (60,61). The effect of CTCF is modulated by its binding partner cohesin, which symmetrises the nucleosome arrays when it co-binds with CTCF (Figure 5D).

The asymmetric nucleosome-depleted regions near CTCF resemble the pattern observed near TSS, and the corresponding effect of NRL decrease as the gene activity increases (Supplementary Figure S14). Importantly, this effect is observed even for CTCF sites that are separated by more than 10,000 bp from the nearest annotated TSS (Supplementary Figure S6). Thus, the effects reported here are not directly related to gene transcription by Pol II. However, they may be linked to transcription of transposons such as Pol III-dependent SINE repeats. Several publications suggested an important role of transposons in the evolution of CTCF sites (62–66), and also it is known that mouse SINE B2 repeats can act as insulators (domain boundaries) *per se* (67). In addition, our data suggests that CTCF may play active role in transposon functioning as transcribed units separating nucleosome arrays. This is in line with recent reports about transcribed transposons associated with CTCF sites (68). Interestingly, previous publications reported that TFIIIC binds to RNA Pol III at tRNA genes and acts as a barrier against the spreading of heterochromatin (69) – this barrier function can be now re-interpreted in light of our results on the association of CTCF with Pol III as well as Pol II outside of gene promoters (Figure 5B). The importance of repetitive DNA sequences in the formation of chromatin boundaries near CTCF is further strengthened by the possibility of non-consensus TF binding in these regions (70). Unexpectedly, the effect of CTCF on the expression of RNA from adjacent locations is short-range repression, which becomes stronger as CTCF binding increases (Figure 6E).

We also showed that the asymmetry of the nucleosome signatures depends on the DNA-defined strength of CTCF binding and may be in addition determined by the CTCF/BORIS competition, because ‘weak’ CTCF binding sites are enriched with the CTCFL recognition motif (Figure 6). BORIS has been previously proposed to interfere with CTCF binding (71), and our results further substantiate its role in the ‘CTCF code’ (43) that defines differential CTCF/BORIS binding.

Secondly, we found that the NRL decrease near CTCF is correlated with CTCF-DNA binding affinity (Figures 1D, F and 5F). This result goes significantly beyond previous observations that the CTCF binding strength is related to a more regular nucleosome ordering near its binding site (44,72) and may have direct functional implications. Strikingly, the variation of NRL as a function of CTCF binding affinity can be as large as ∼20 bp (the difference between NRL near the weakest CTCF-like motifs and the strongest CTCF-bound sites). Cohesin has a similar effect, but it is pronounced only when cohesin co-binds with CTCF. None of other DNA-binding proteins showed such behaviour (Figure 2). This uniqueness of CTCF can be explained by the large variability of its binding affinity through different combinations of its 11 zinc fingers that allows creating a ‘CTCF code’ (61,71,73). The effect of the NRL dependence on CTCF binding strength is most profound 3′-downstream of CTCF motifs, where it can be approximated by a linear function (Figure 5F). This strong nucleosome patterning downstream but not upstream of CTCF is comparable to that of transcription start sites (TSSs) of protein-coding genes. In analogy, this effect could provide an additional argument that this may be linked to the transcription of non-coding repeats enclosing CTCF including Pol III-dependent SINEs.

Thus, our data suggests that the nucleosome arrangement near CTCF is defined by an active, remodeller-dependent process. Therefore, we analysed the contributions to this process by each of 8 chromatin remodellers that have been experimentally profiled in ESCs (Figure 4). We found that Snf2h has a major role in NRL decrease near CTCF, consistent with previous studies of Snf2H knockout in HeLa cells (74) and ESCs (40). In accord with the latter study, we observed that BRG1 has no detectable effect on NRL near CTCF, although it may be still involved in nucleosome positioning near TAD boundaries (75). Our investigation also identified Chd8 and EP400 as regulators of NRL near CTCF (Figure 4B, Supplementary Figure S10). These findings are consistent with the previous investigations that showed that Chd8 physically interacts with CTCF and knockdown of Chd8 abolishes the insulator activity of CTCF sites required for IGF2 imprinting (76). One can hypothesise that this kind of insulator activity of CTCF is related to the boundary created by the nucleosome-free region 5′-upstream of the CTCF motif reported here, which may physically prevent the spreading of DNA methylation and other epigenetic modifications. Interestingly, our analysis revealed that the main chromatin remodeller responsible for the asymmetry of the nucleosome array near CTCF is Chd4. We show that Chd4 is both the top CTCF-associated remodeller (Figure 4A) and the sole remodeller responsible for the CTCF-dependent nucleosome occupancy peak 3′-downstream of the CTCF motif (Figure 5C). This finding may be important in the context of recent studies indicating that Chd4 is increasing the nucleosome density at regulatory regions (77).

The third major finding of this work concerns the effects of CTCF motif directionality and binding strength on nucleosome rearrangement during cell differentiation. Our calculations showed that the binding affinity is a good predictor for a given CTCF site being preserved upon cell differentiation (Figure 7A). This may be used as a foundation for the ‘CTCF code’ determining its differential binding as the cell progresses along the Waddington-type pathways. A specific subclass of common CTCF sites preserved upon cell differentiation tends to keep a small NRL, while the average NRL near all CTCF sites increases due to the active nucleosome repositioning 3′-downstream of CTCF motifs (Figure 7). A previous study reported a related distinction of common versus non-common CTCF sites based on the distance between the two nucleosomes downstream and upstream of CTCF (78). The preservation of NRL for common CTCF sites may give rise to a new effect where differential CTCF binding defines extended regions which do not change (or change minimally) their nucleosome positioning. Unexpectedly, the nucleosome-depleted region 5′-upstream of the CTCF motif remains even after CTCF depletion from a given site during differentiation. These nucleosome-depleted regions can have important functional roles, including the preservation of chromatin states while CTCF-dependent loops are dynamic and frequently break and reform throughout the cell cycle (79). For example, if the spreading of some chemical modifications of DNA or histones along the genomic coordinate requires enzymes cooperatively binding to the adjacent nucleosomes, then the consistent lack of a nucleosome at a given location can stop the propagation of the ‘epigenetic wave’.

Finally, our finding of the asymmetry of CTCF-dependent chromatin boundaries at the scale of several nucleosomes may provide the missing mechanistic explanation for the asymmetry of chromatin boundaries at the scale of hundreds to thousands of nucleosomes reported recently (80,81). As we showed, TAD boundaries often contain several directed CTCF motifs (Figure 7F, Supplementary Figure S18). One can speculate that in this case the effects of individual CTCF sites accumulate, leading to the formation of a specific, asymmetric and 3D-structured nucleosome organisation at TAD boundary (schematically represented in Figure 8C). Such additivity of individual CTCF motifs could explain previous observations where the removal of part of the DNA sequence responsible for the boundary does not lead to the complete loss of TAD insulation (82). In general, the asymmetric nucleosome organisation near CTCF reported here can be particularly interesting in light of the ongoing debate on the functional roles of chromatin boundaries in gene regulation.

## DATA AVAILABILITY

Our software is available at https://github.com/chrisclarkson/NRLcalc

## Supplementary Material

gkz908_Supplemental_File

## Notes

Present address: Ralph Samarista, Department of Biological and Medical Sciences, Oxford Brookes University, Headington Campus, Oxford OX3 0BP, UK.
