# Supplementary material for: CTCF-dependent chromatin boundaries formed by asymmetric nucleosome arrays with decreased linker length
Source: Nucleic Acids Res. 2019 Oct 30;47(21):11181–96. doi: 10.1093/nar/gkz908 (PMC6868436; doi:10.1093/nar/gkz908)
Supplement: gkz908_Supplemental_File [file gkz908_supplemental_file.pdf]

Supplementary Materials

CTCF-dependent chromatin boundaries formed by asymmetric nucleosome arrays with decreased linker length

Christopher T. Clarkson[1], Emma A. Deeks[1,2], Ralph Samarista[1,3], Hulkar Mamayusupova[1], Victor B. Zhurkin[4] and Vladimir B. Teif[1,*]

[1] School of Biological Sciences, University of Essex, Wivenhoe Park, Colchester, CO4 3SQ, UK

[2] Biological Sciences BSc Program, University of Essex, Wivenhoe Park, Colchester, CO4 3SQ, UK

[3] Wellcome Trust Vacation Student. Current address: Department of Biological and Medical Sciences, Oxford Brookes University, Headington Campus, Oxford, OX3 0BP, UK

[4] Center for Cancer Research, National Cancer Institute, National Institutes of Health, Bethesda, MD 20892, USA

* Correspondence should be addressed to Vladimir B. Teif: Phone: E-mail: vteif@essex.ac.uk

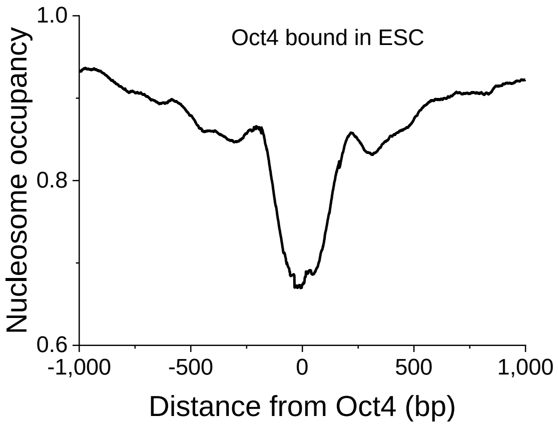

Figure S1. Average nucleosome occupancy profiles in ESC around bound Oct4. ChIP-seq data for Oct4 is from (1) and MNase-seq data is from (2).

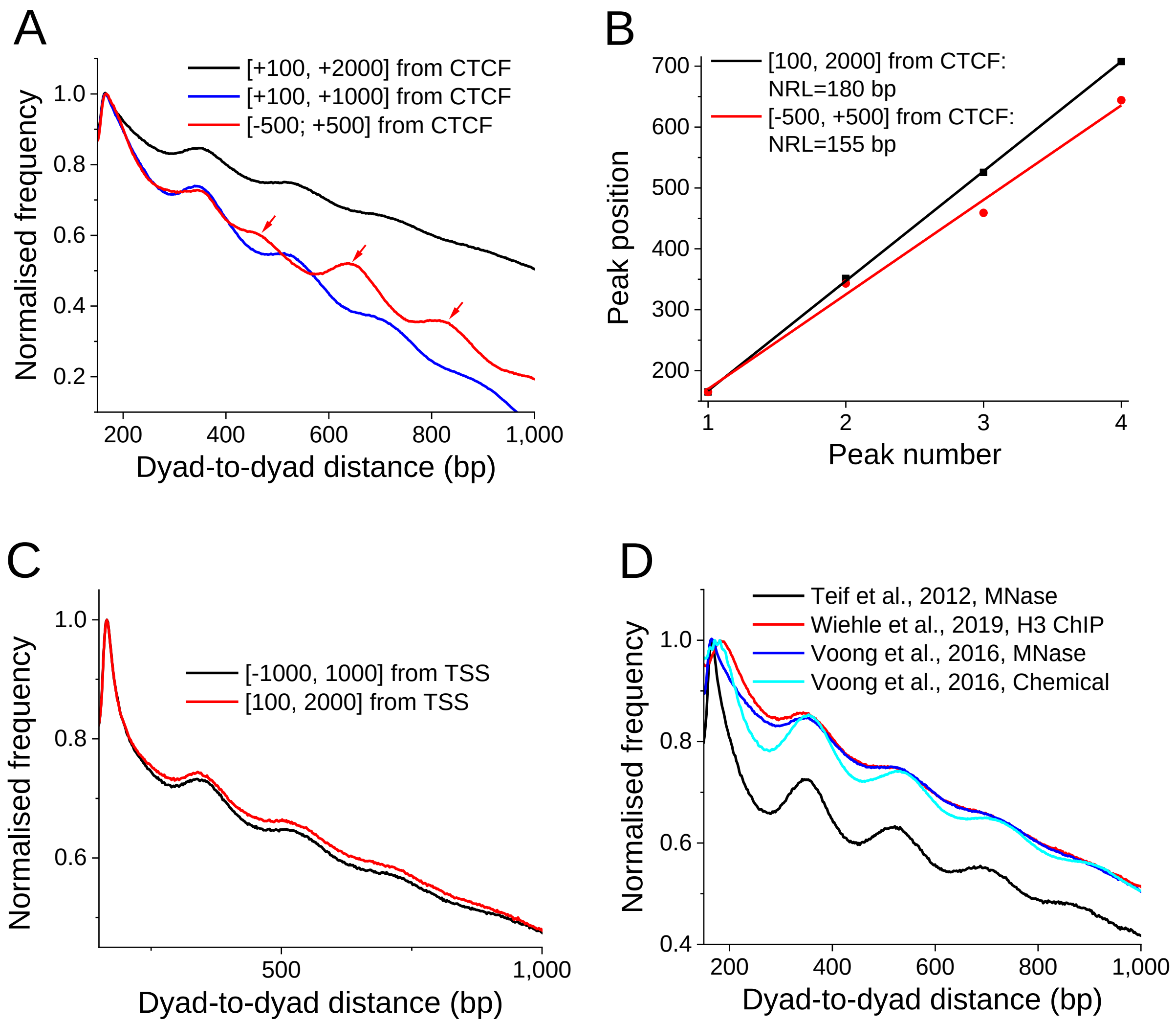

Figure S2. The effect of region location near CTCF on the apparent NRL value. A) Phasograms depicting the normalised frequency of nucleosome dyad-to-dyad distances calculated using NucTools for three different regions near CTCF sites: [100, 2000], [100, 1000] and [-500, 500]. Both [100, 2000] and [100, 1000] patterns oscillate with NRL=174. In the case of the [-500, 500] phasogram additional peaks (indicated by red arrows) appear which correspond to distances between nucleosomes on different sides of CTCF, thus resulting in an apparent NRL<160. B) NRLs calculated from the phasograms shown in panel (A). Region [-500, 500] is characterised by an unrealistically small NRL=155bp which is an artefact of the interference of two waves of distances between nucleosomes located on different sides of CTCF. C) Comparison of the phasograms showing the normalised frequency of nucleosome dyad-to-dyad distances for the regions [-1000, 1000] and [100, 2000] from TSS. The NRLs calculated based on these phasograms are not significantly different (172+/-1 bp vs. 168+/-3 bp respectively). D) Phasograms calculated for the region [100, 2000] near CTCF site for two different MNase-seq datasest from (3) and (2), MNase-assisted H3 ChIP-seq from (4) and chemical mapping from (2).

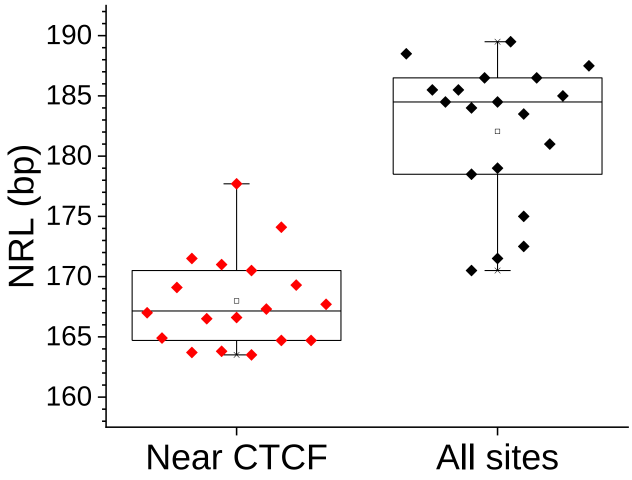

Figure S3. NRLs calculated near binding sites of 18 stemness-related chromatin proteins in ESCs in the region [250, 1000] from the TF sites. The same nucleosome positioning and TF-binding datasets as in Figure 1C are used. Left: TF binding sites in the vicinity of CTCF; right: all TF binding sites irrespective of their distance from CTCF.

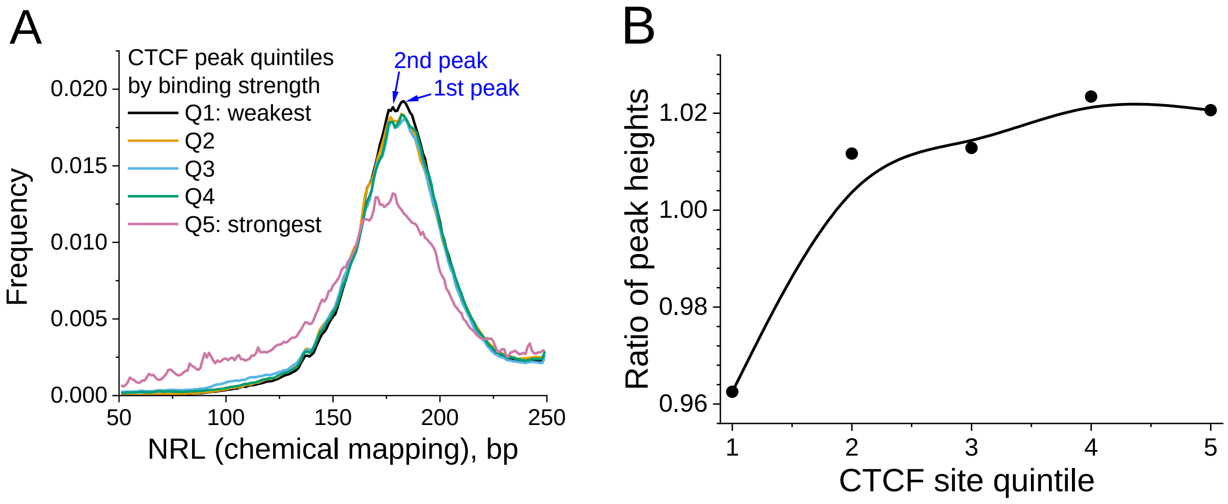

Figure S4. A) A histogram of nucleosome dyad-to-dyad distances determined using chemical mapping for different CTCF quintiles defined based on CTCF binding strength determined by the height of ChIP-seq CTCF peaks. Note that the dyad-to-dyad distances determined using chemical mapping cannot be directly compared to MNase-seq based NRLs due to an inherent bias of the chemical mapping experimental setup that we discussed previously (5). B) The ratio between heights of 2nd peak and 1st peak of the distribution of lengths of chemical mapping-based dyad-to-dyad distances shown in panel (A) as a function of the CTCF site quintile based on the heights of CTCF ChIP-seq peaks.

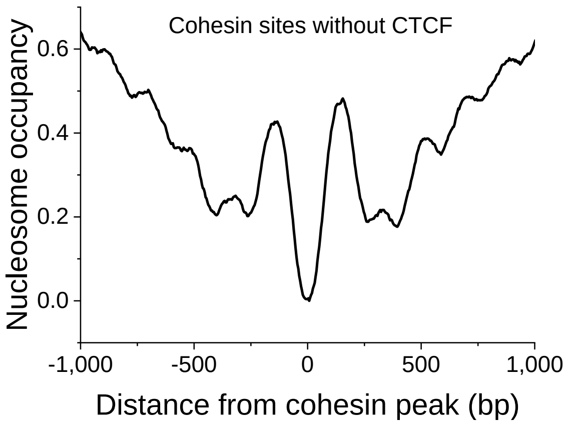

Figure S5. Average nucleosome occupancy profiles around sites bound by cohesin in ESC, taking into account only sites that do not contain CTCF motifs.

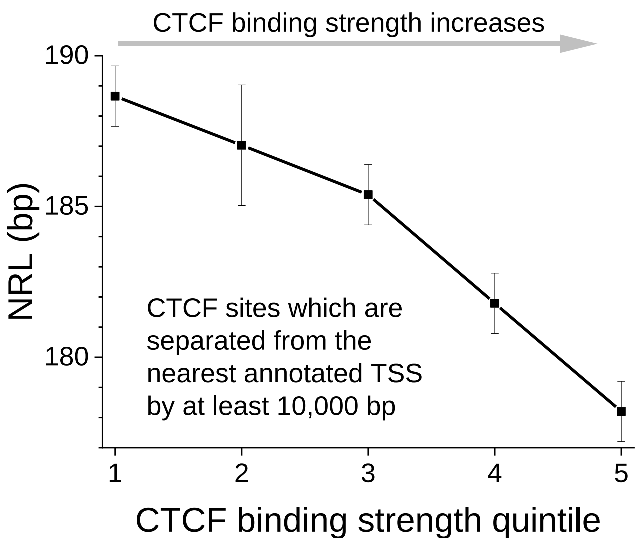

Figure S6. Dependence of NRL in the region [100, 2000] near CTCF on the strength of CTCF binding, excluding the effect of promoters. This calculation used only CTCF sites separated from the nearest TSS by at least 10,000 bp. The error bars show standard deviation.

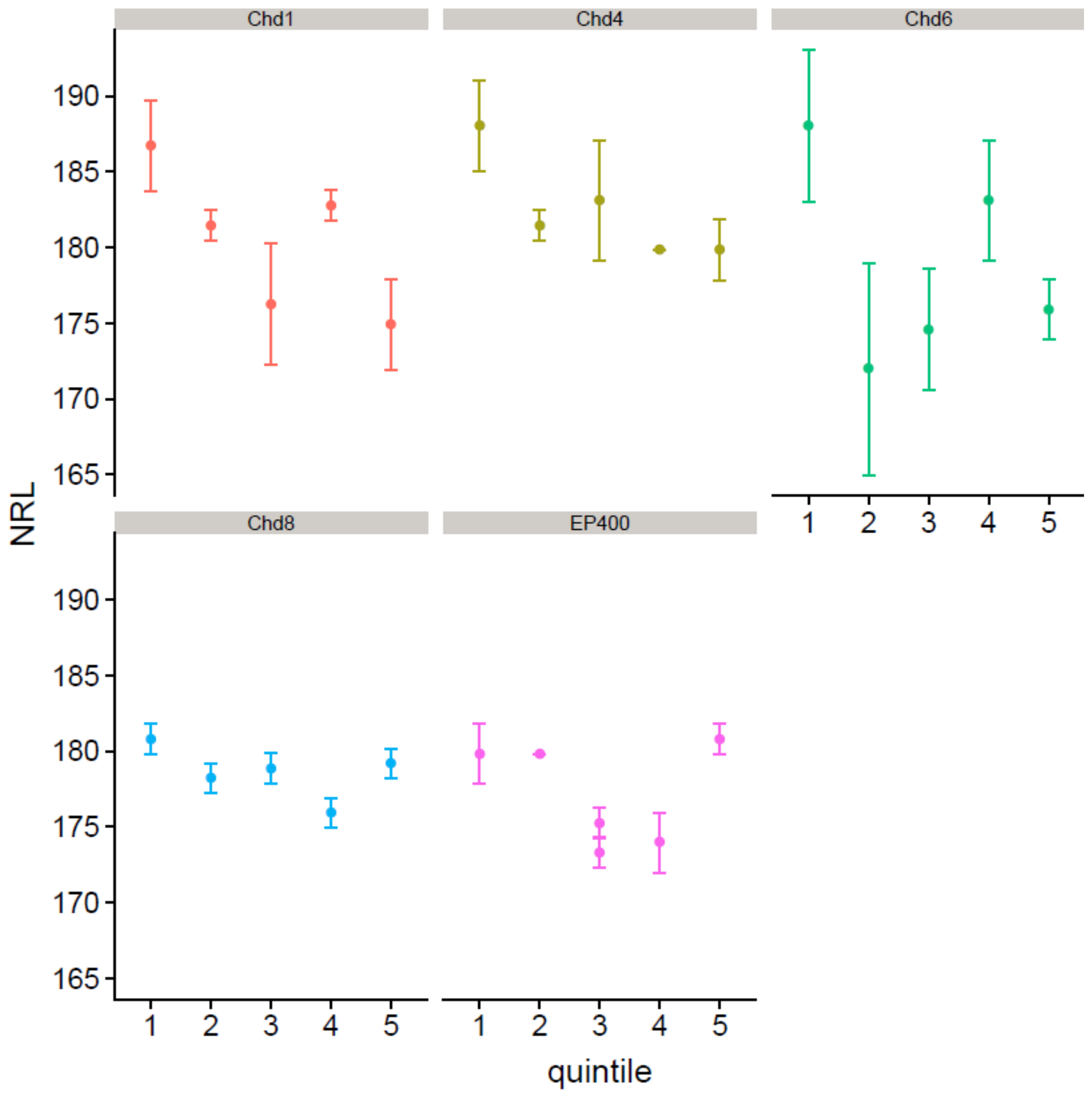

Figure S7. NRL calculated in the region [-1000, 1000] from the summits of ChIP-seq peaks of chromatin remodellers Chd1, Chd4, Chd6, Chd8, EP400 in ESCs.

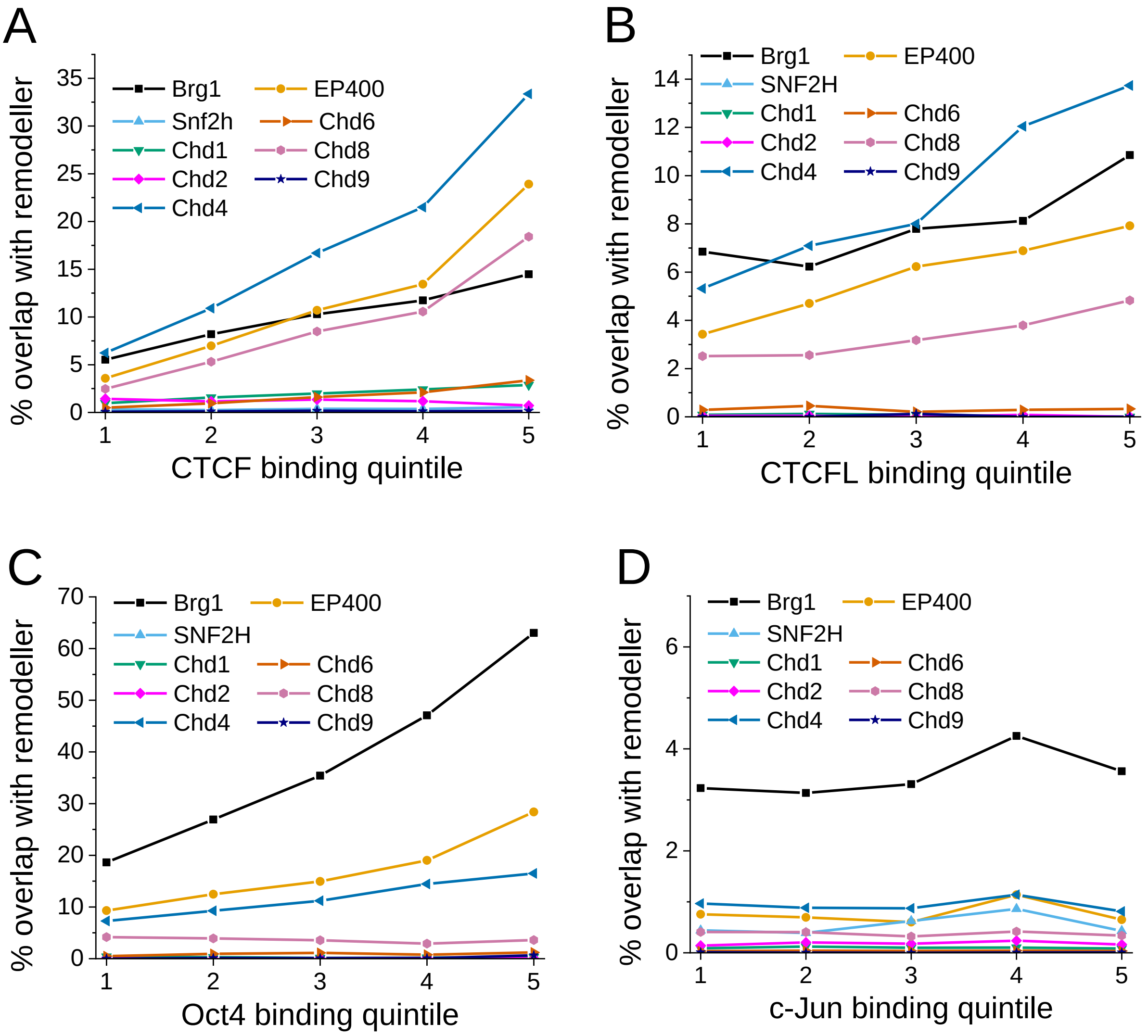

Figure S8. Overlapping of chromatin remodellers with different TFs. The percentage of overlap of a given TF with a given remodeller was defined as the ratio of TF sites overlapping with ChIP-seq peaks of a given remodeller to the total number of CTCF sites in a given quintile. Binding sites of each TF were split into quintiles according to their predicted binding strengths. A) CTCF, B) CTCFL (BORIS), C) Oct4, D) c-Jun.

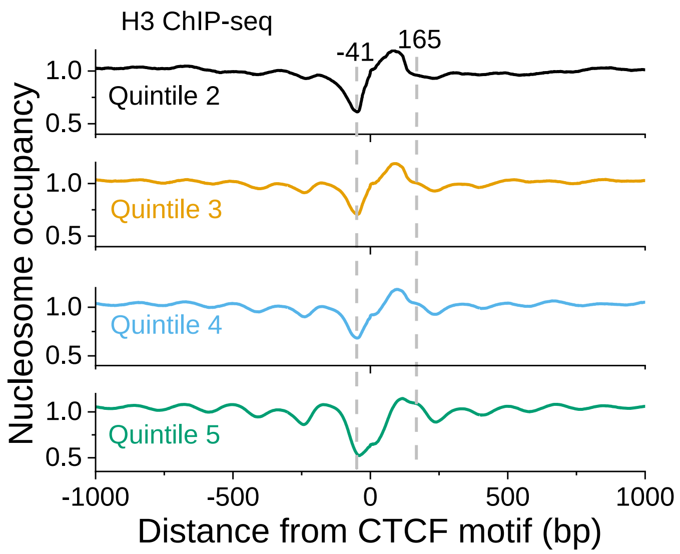

Figure S9. Aggregate nucleosome occupancy profiles around directional CTCF as in Figure 5, calculated using MNase-assisted histone H3 ChIP-seq signal from (Wiehle et al., 2019).

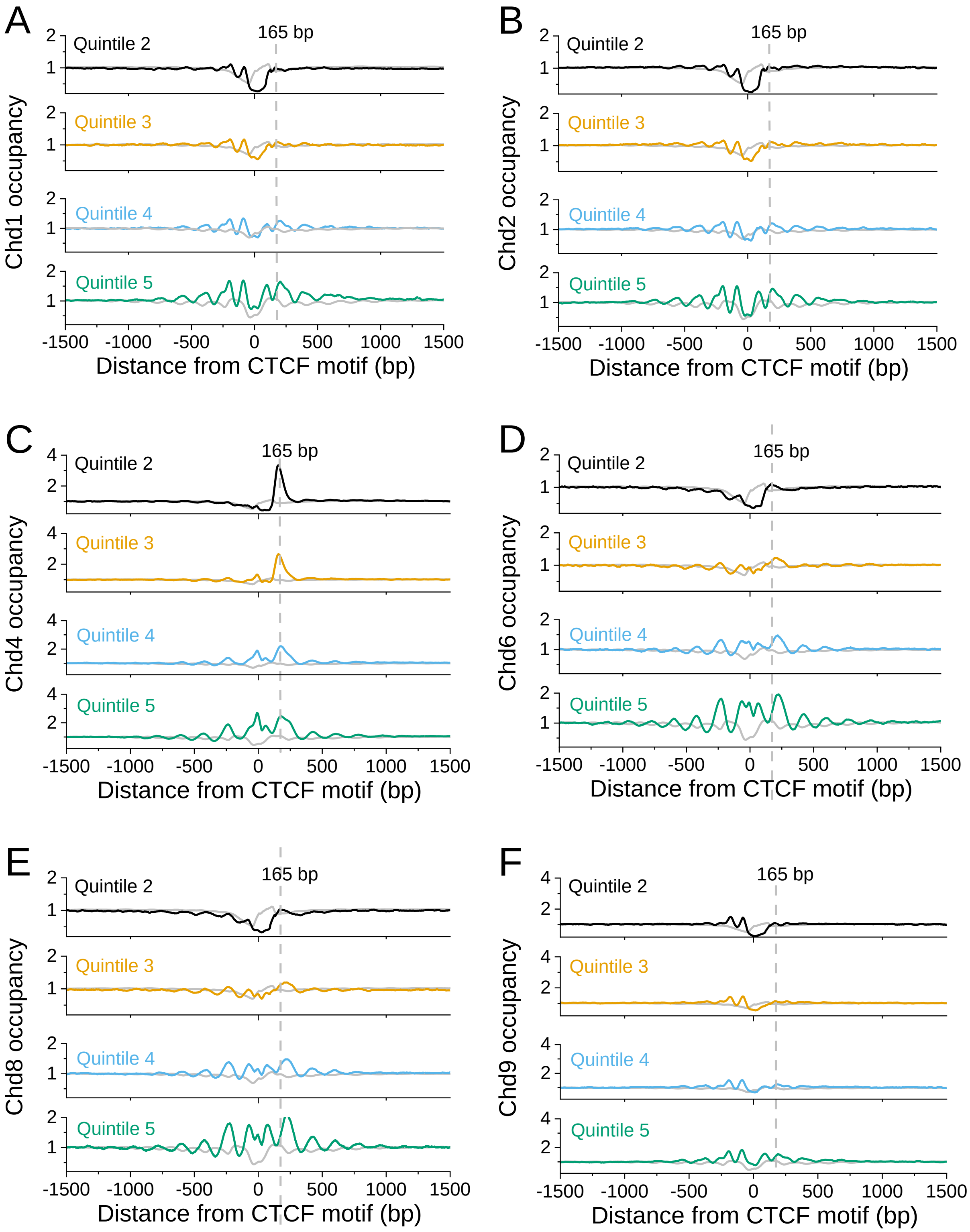
A
B
C
D
E
F
165 bp
Quintile 2
Quintile 3
Quintile 4
Quintile 5
Chd1 occupancy
Chd2 occupancy
Chd4 occupancy
Chd6 occupancy
Chd8 occupancy
Chd9 occupancy
-1500 -1000 -500 0 500 1000 1500
Distance from CTCF motif (bp)

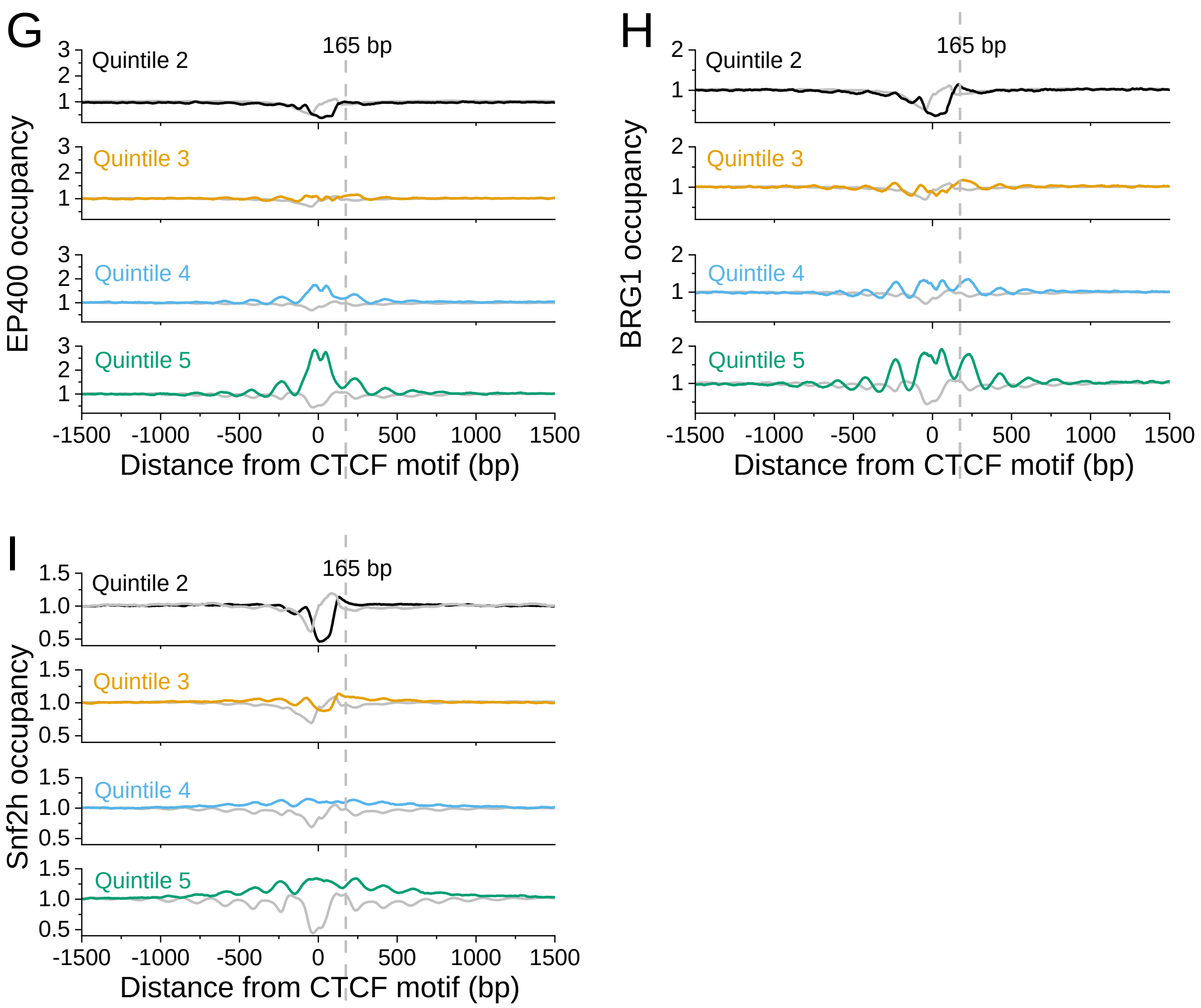

Figure S10. Aggregate profiles of the occupancy of chromatin remodellers near directional CTCF motifs in ESCs. Aggregate occupancy profiles for nine chromatin remodellers around all predicted CTCF motifs in the mouse genome show that only Chd4 has a CTCF-dependent peak at 165 bp (note a different scale for Chd4). Remodeller profiles are aligned around CTCF motifs split into quintiles with increasing CTCF binding strength as follows: black – 2nd quintile; orange – 3rd quintile; blue – 4th quintile; green – 5th quintile. Grey solid lines show the corresponding nucleosome occupancy based on MNase-seq as in Figure 5.

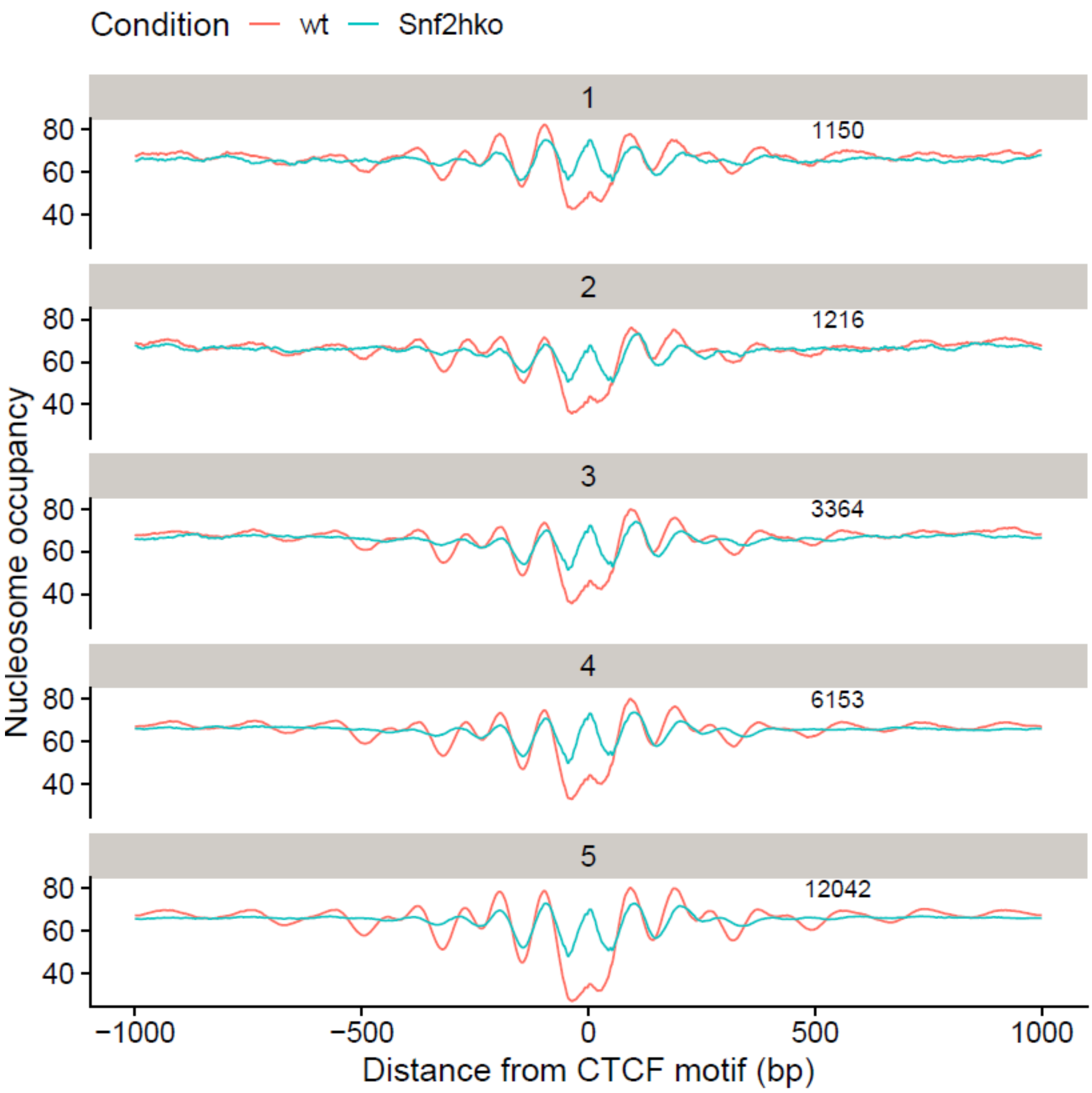

Figure S11. Aggregate profiles of nucleosome occupancy around CTCF sites lost upon Snf2h knockout. Red lines – wild type ESCs, blue lines – Snf2h knockout. The calculations are performed based on MNase-seq and CTCF ChIP-seq data from (6). The CTCF motifs predicted in the mouse genome with up to 80% similarity score were split into 5 quintiles as described in Methods, and intersected with CTCF sites lost upon Snf2h knockout (6). The numbers of CTCF motifs remaining in each quintile upon this intersection are indicated on the figure.

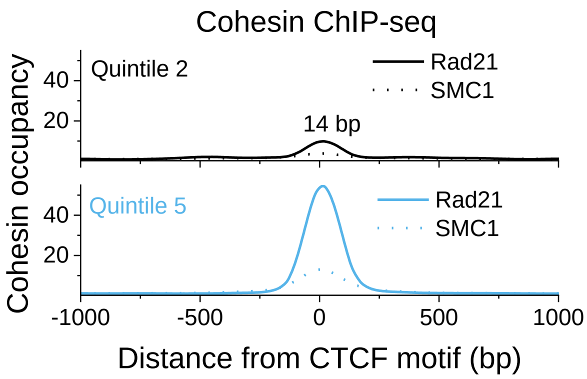

Figure S12. Aggregate profiles of cohesin subunits SMC1 and Rad21 measured by ChIP-seq in ESC, around directional CTCF motifs. The peak of Rad21 is shifted 14 bp from the centre of CTCF motif while the peak of SMC1 coincides with the centre of CTCF motif.

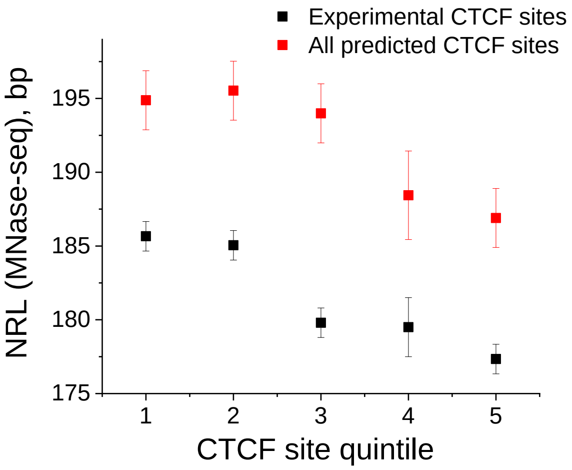

Figure S13. NRL in the region [300, 2000] near CTCF as a function of CTCF binding strength. The effect of NRL decrease with increase of CTCF binding strength remains even after excluding the CTCF-dependent nucleosome at +165 bp and performing NRL calculation for the region [300, 2000] downstream of CTCF sites (to the right from CTCF using plus strand coordinates).

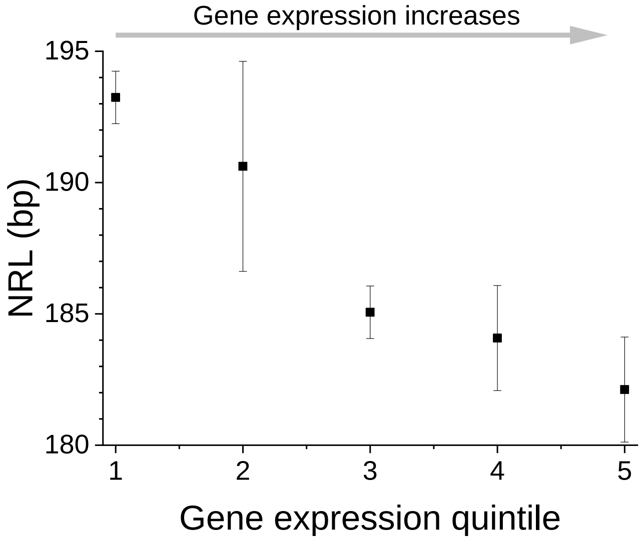

Figure S14. NRL in the region [-1000, 1000] near TSS as a function of gene expression. Genes have been split into 5 quintiles according to their normalised expression levels reported in (4).

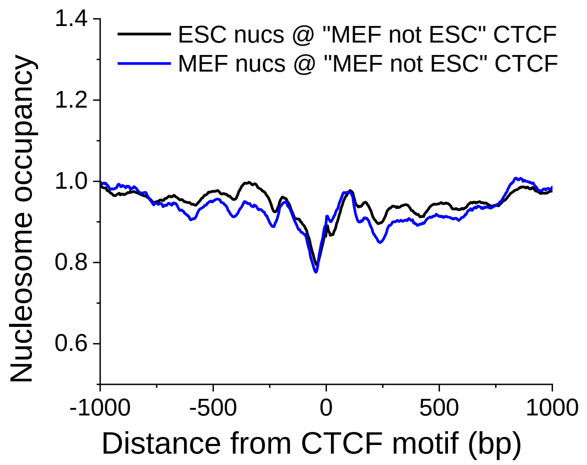

Figure S15. Effects of CTCF-dependent boundary directionality in stem cell differentiation. Nucleosome occupancy in ESCs (black) and MEFs (blue) around CTCF sites “MEF not ESC” that are present in MEFs but not in ESCs, calculated taking into account CTCF motif directionality.

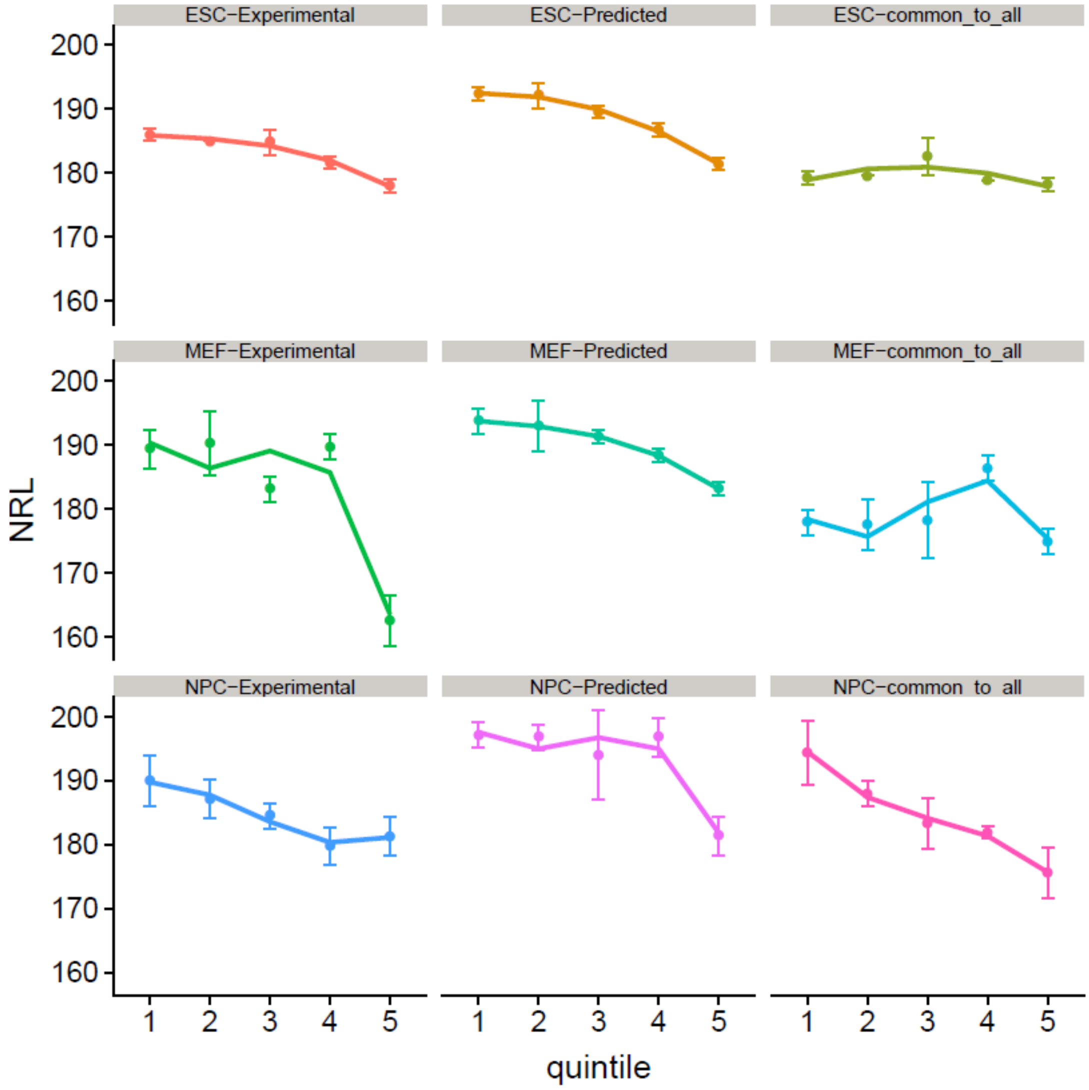

Figure S16. Effect of ESC differentiation on NRL. NRL values are calculated as a function of the CTCF site quintile. Top row: NRLs calculated based on the MNase-seq dataset in ESCs from Teif et al., 2012 for all experimental CTCF sites in ESCs determined in Shen et al, 2012 (left), all computationally predicted CTCF sites (middle) and common CTCF sites that have been determined experimentally in each of ESCs, NPCs and MEFs (right). Middle row: NRLs calculated based on the MNase-seq dataset in MEFs from Teif et al., 2012 for all experimental CTCF sites in MEF determined in Shen et al, 2012 (left), all computationally predicted CTCF sites (middle) and common CTCF sites that have been determined experimentally in each of ESCs, NPCs and MEFs (right). Bottom row: NRLs calculated based on the MNase-seq dataset in NPCs from Teif et al., 2012 for all experimental CTCF sites in NPCs determined in Bonev et al., 2017 (left), all computationally predicted CTCF sites (middle) and common CTCF sites that have been determined experimentally in each of ESCs, NPCs and MEFs (right).

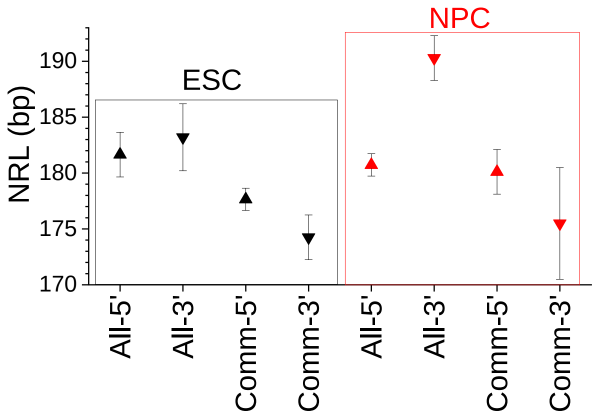

Figure S17. Effect of CTCF directionality on NRL preservation in ESC differentiation. NRLs in region [100, 2000] from CTCF's binding motifs overlapping with experimentally confirmed CTCF binding sites were calculated separately 5'-upstream and 3'-downstream of CTCF motifs in ESCs and NPCs. The major NRL change during differentiation is in the region 3'-downstream of CTCF motifs.

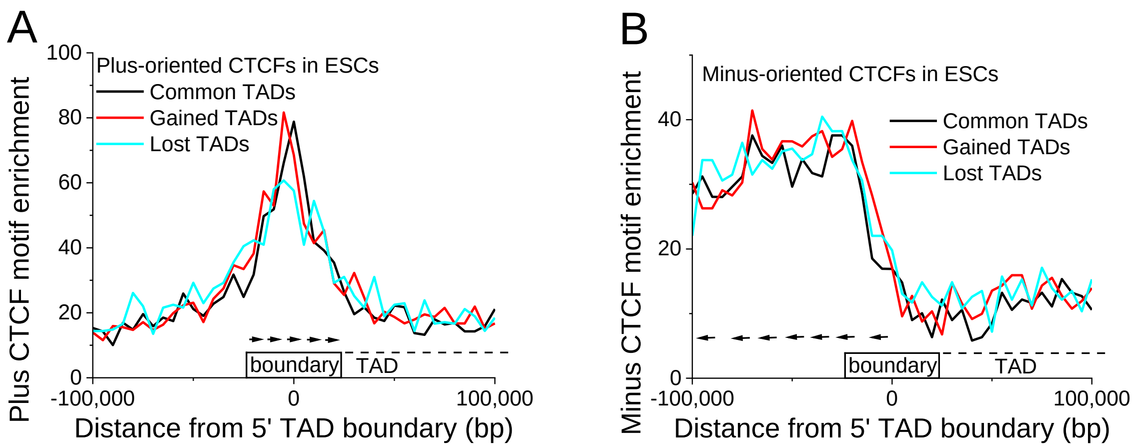

Figure S18. Enrichment of CTCF motifs bound by CTCF in ESCs near TAD boundaries common to ESC and NPC (black line), TAD boundaries not present in ESC but gained in NPCs (red) and TAD boundaries present in ESC but lost in NPC (light blue line). The arrows demonstrate directions of CTCF motifs.

Supplemental Table ST1. Summary of experimental datasets used in this study

Name	Accession #	Literature reference	Figure #
Nanog in ESC	GSM3123484	(7)	1C, S3
Oct4 in ESC	GSM2417142	(1)	1C, S1, S3
Sox2 in ESC	GSM2417143	(1)	1C, S3
Klf4 in ESC	GSM2417144	(1)	1C, S3
cMyc in ESC	GSM2417145	(1)	1C, S3
Esrrb in ESC	GSM2561449	(8)	1C, S3
GATA1 in ESC	GSM453997	(9)	1C, S3
Ep300 in ESC	ENCSR000CCD GSM918750	(10)	1C, S3
Tal1 in ESC	ENCSR000DIN GSM923579	(10)	1C, S3
Zbtb2 in ESC	GSM2716083	(11)	1C, S3
ZNF384 in ESC	ENCSR000ERV GSM1003807	(10)	1C, S3
CTCF in ESC and MEF	ENCSR000CCB GSM918748	(10)	1C, S3
STAT3 in ESC	GSM288353	(10)	1C, S3
GATA2 in ESC	ENCSR000DIE GSM923587	(10)	1C, S3
cJun in ESC	GSM1587320	(12)	1C, S3
Max in ESC	GSM1171650	(13)	1C, S3
Arid3a in ESC	GSM1370509	(14)	1C, S3
Sox17 in ESC	GSM1059856	(15)	1C, S3
CpG islands in the mouse genome	Obtained from authors' web site	(16)	3D
BRG1 in ESC	GSM359413	(17)	3D, 4, S10
Snf2h in ESC	GSE80049	(18)	3D, 4
Nucleosome chemical mapping in ESC	GSE82127	(2)	1C, S2D, S3, S4
MNase-seq in ESC (Voong et al., 2016)	GSM2183911	(2)	1A, 1B, 1D, 2, 3, 4, 5, 6, S2, S8, S9, S11
MNase-seq in ESC, NPC, MEF (Teif et al, 2012, 2014)	GSE40896	(3,19)	7, S2D, S15-18

MNase-assisted ChIP-seq against histone H3 in ESC; RNA-seq	GSE114599	(4)	S2D, S9
BRG1 in ESC	GSE64825	(20)	3D, 4
Chd1 in ESC	GSE64825	(20)	3D, 4, S8, S10
Chd2 in ESC	GSE64825	(20)	3D, 4, S8, S10
Chd4 in ESC	GSE64825	(20)	3D, 4, 5C, S8, S10
Chd6 in ESC	GSE64825	(20)	3D, 4, S8, S10
Chd8 in ESC	GSE64825	(20)	3D, 4, S8, S10
Chd9 in ESC	GSE64825	(20)	3D, 4, S8, S10
EP400 in ESC	GSE64825	(20)	3D, 4, S8, S10
Coordinates of chromatin loops and TADs in ESCs and NPCs	TAD coordinates in mm10 were obtained from Table S2. BED files with the loop coordinates in mm10 were provided by the authors.	(21)	3D, 7F, S18
Gene promoters	RefSeq	(22)	3D, S14
Annotated TSSs	Genomatix Eldorado		S6
Gene enhancers	http://fantom.gsc.riken.jp (all permissive)	(23)	3D
CTCF in ESC and MEF	GSE114599	(4)	6
CTCF in ESC and MEF	GSE27944	(24)	6
CTCF in ESC and NPC	GSE96107	(21)	6
SMC1 in ESC	GSM3175702	(25)	5D, S5, S12
RAD54 in ESC	GSE81676	(26)	S12
MNase-seq and CTCF ChIP-seq in WT ESCs and Snf2h KO ESCs	GSE112136	(6)	S11

Supplementary references

1. Chronis, C., Fiziev, P., Papp, B., Butz, S., Bonora, G., Sabri, S., Ernst, J. and Plath, K. (2017) Cooperative Binding of Transcription Factors Orchestrates Reprogramming. *Cell*, **168**, 442-459 e420.
2. Voong, L.N., Xi, L., Sebeson, A.C., Xiong, B., Wang, J.P. and Wang, X. (2016) Insights into Nucleosome Organization in Mouse Embryonic Stem Cells through Chemical Mapping. *Cell*, **167**, 1555-1570 e1515.
3. Teif, V.B., Vainshtein, Y., Caudron-Herger, M., Mallm, J.P., Marth, C., Höfer, T. and Rippe, K. (2012) Genome-wide nucleosome positioning during embryonic stem cell development. *Nat Struct Mol Biol*, **19**, 1185-1192.
4. Wiehle, L., Thorn, G.J., Raddatz, G., Clarkson, C.T., Rippe, K., Lyko, F., Breiling, A. and Teif, V.B. (2019) DNA (de)methylation in embryonic stem cells controls CTCF-dependent chromatin boundaries. *Genome Res*, **29**, 750-761.
5. Vainshtein, Y., Rippe, K. and Teif, V.B. (2017) NucTools: analysis of chromatin feature occupancy profiles from high-throughput sequencing data. *BMC Genomics*, **18**, 158.
6. Barisic, D., Stadler, M.B., Iurlaro, M. and Schubeler, D. (2019) Mammalian ISWI and SWI/SNF selectively mediate binding of distinct transcription factors. *Nature*, **569**, 136-140.
7. Kim, K.Y., Tanaka, Y., Su, J., Cakir, B., Xiang, Y., Patterson, B., Ding, J., Jung, Y.W., Kim, J.H., Hysolli, E. *et al.* (2018) Uhrf1 regulates active transcriptional marks at bivalent domains in pluripotent stem cells through Setd1a. *Nature communications*, **9**, 2583.
8. Xie, L., Torigoe, S.E., Xiao, J., Mai, D.H., Li, L., Davis, F.P., Dong, P., Marie-Nelly, H., Grimm, J., Lavis, L. *et al.* (2017) A dynamic interplay of enhancer elements regulates Klf4 expression in naive pluripotency. *Genes Dev*, **31**, 1795-1808.
9. Chen, X., Xu, H., Yuan, P., Fang, F., Huss, M., Vega, V.B., Wong, E., Orlov, Y.L., Zhang, W., Jiang, J. *et al.* (2008) Integration of external signaling pathways with the core transcriptional network in embryonic stem cells. *Cell*, **133**, 1106-1117.
10. Shen, Y., Yue, F., McCleary, D.F., Ye, Z., Edsall, L., Kuan, S., Wagner, U., Dixon, J., Lee, L., Lobanenkov, V.V. *et al.* (2012) A map of the cis-regulatory sequences in the mouse genome. *Nature*, **488**, 116-120.
11. Karemaker, I.D. and Vermeulen, M. (2018) ZBTB2 reads unmethylated CpG island promoters and regulates embryonic stem cell differentiation. *EMBO Rep*, **19**.
12. Liu, J., Han, Q., Peng, T., Peng, M., Wei, B., Li, D., Wang, X., Yu, S., Yang, J., Cao, S. *et al.* (2015) The oncogene c-Jun impedes somatic cell reprogramming. *Nat Cell Biol*, **17**, 856-867.
13. Krepelova, A., Neri, F., Maldotti, M., Rapelli, S. and Oliviero, S. (2014) Myc and max genome-wide binding sites analysis links the Myc regulatory network with the polycomb and the core pluripotency networks in mouse embryonic stem cells. *PLoS One*, **9**, e88933.
14. Rhee, C., Lee, B.-K., Beck, S., Anjum, A., Cook, K.R., Popowski, M., Tucker, H.O. and Kim, J. (2014) Arid3a is essential to execution of the first cell fate decision via direct embryonic and extraembryonic transcriptional regulation. *Genes & Development*, **28**, 2219-2232.

15. Aksoy, I., Jauch, R., Chen, J., Dyla, M., Divakar, U., Bogu, G.K., Teo, R., Leng Ng, C.K., Herath, W., Lili, S. *et al.* (2013) Oct4 switches partnering from Sox2 to Sox17 to reinterpret the enhancer code and specify endoderm. *EMBO J*, **32**, 938-953.
16. Irizarry, R.A., Wu, H. and Feinberg, A.P. (2009) A species-generalized probabilistic model-based definition of CpG islands. *Mamm Genome*, **20**, 674-680.
17. Ho, L., Jothi, R., Ronan, J.L., Cui, K., Zhao, K. and Crabtree, G.R. (2009) An embryonic stem cell chromatin remodeling complex, esBAF, is an essential component of the core pluripotency transcriptional network. *Proc Natl Acad Sci U S A*, **106**, 5187-5191.
18. Local, A., Huang, H., Albuquerque, C.P., Singh, N., Lee, A.Y., Wang, W., Wang, C., Hsia, J.E., Shiau, A.K., Ge, K. *et al.* (2018) Identification of H3K4me1-associated proteins at mammalian enhancers. *Nat Genet*, **50**, 73-82.
19. Teif, V.B., Beshnova, D.A., Vainshtein, Y., Marth, C., Mallm, J.P., Höfer, T. and Rippe, K. (2014) Nucleosome repositioning links DNA (de)methylation and differential CTCF binding during stem cell development. *Genome Res*, **24**, 1285-1295.
20. de Dieuleveult, M., Yen, K., Hmitou, I., Depaux, A., Boussouar, F., Bou Dargham, D., Jounier, S., Humbertclaude, H., Ribierre, F., Baulard, C. *et al.* (2016) Genome-wide nucleosome specificity and function of chromatin remodellers in ES cells. *Nature*, **530**, 113-116.
21. Bonev, B., Mendelson Cohen, N., Szabo, Q., Fritsch, L., Papadopoulos, G.L., Lubling, Y., Xu, X., Lv, X., Hugnot, J.P., Tanay, A. *et al.* (2017) Multiscale 3D Genome Rewiring during Mouse Neural Development. *Cell*, **171**, 557-572 e524.
22. Pruitt, K.D., Brown, G.R., Hiatt, S.M., Thibaud-Nissen, F., Astashyn, A., Ermolaeva, O., Farrell, C.M., Hart, J., Landrum, M.J., McGarvey, K.M. *et al.* (2014) RefSeq: an update on mammalian reference sequences. *Nucleic Acids Res*, **42**, D756-763.
23. Lizio, M., Harshbarger, J., Shimoji, H., Severin, J., Kasukawa, T., Sahin, S., Abugessaisa, I., Fukuda, S., Hori, F., Ishikawa-Kato, S. *et al.* (2015) Gateways to the FANTOM5 promoter level mammalian expression atlas. *Genome Biol*, **16**, 22.
24. Martin, D., Pantoja, C., Fernandez Minan, A., Valdes-Quezada, C., Molto, E., Matesanz, F., Bogdanovic, O., de la Calle-Mustienes, E., Dominguez, O., Taher, L. *et al.* (2011) Genome-wide CTCF distribution in vertebrates defines equivalent sites that aid the identification of disease-associated genes. *Nat Struct Mol Biol*, **18**, 708-714.
25. Sun, F., Chronis, C., Kronenberg, M., Chen, X.F., Su, T., Lay, F.D., Plath, K., Kurdistani, S.K. and Carey, M.F. (2019) Promoter-Enhancer Communication Occurs Primarily within Insulated Neighborhoods. *Mol Cell*, **73**, 250-263 e255.
26. Kim, H.S., Tan, Y., Ma, W., Merkurjev, D., Destici, E., Ma, Q., Suter, T., Ohgi, K., Friedman, M., Skowronska-Krawczyk, D. *et al.* (2018) Pluripotency factors functionally premark cell-type-restricted enhancers in ES cells. *Nature*, **556**, 510-514.
